# Development of the MDMA-Assisted Psychotherapy Side Effects Tool (M-SET): a Delphi study

**DOI:** 10.1136/bmjopen-2025-105630

**Published:** 2026-05-11

**Authors:** Julia Colcott, Alexandre A Guerin, Olivia Carter, Matthew J Baggott, Anya Bershad, Alicia Danforth, Harriet de Wit, Allison A Feduccia, Matthew G Kirkpatrick, Matthias E Liechti, Peter Oehen, Yasmin Schmid, Gillinder Bedi

**Affiliations:** 1 Melbourne School of Psychological Sciences, University of Melbourne, Melbourne, Victoria, Australia; 2 Centre for Youth Mental Health, University of Melbourne, Melbourne, Victoria, Australia; 3 Orygen, Melbourne, Victoria, Australia; 4 Tactogen, Inc, Palo Alto, California, USA; 5 UCLA Semel Institute for Neuroscience and Human Behavior, Los Angeles, California, USA; 6 The Lundquist Institute for Biomedical Innovation, Harbor-UCLA Medical Center, Torrance, California, USA; 7 Department of Psychiatry and Behavioral Neuroscience, The University of Chicago, Chicago, Illinois, USA; 8 Department of Research Development, MAPS Public Benefit Corp, Santa Cruz, California, USA; 9 University of Southern California Keck School of Medicine, Los Angeles, California, USA; 10 Department of Clinical Research, University Hospital Basel, Basel, Switzerland; 11 Division of Clinical Pharmacology and Toxicology, University Hospital Basel, Basel, Switzerland; 12 Department of Biomedicine, University of Basel, Basel, Switzerland; 13 Private Practice of Psychiatry and Psychotherapy, Biberist, Switzerland

**Keywords:** Adult psychiatry, Adverse events, PSYCHIATRY

## Abstract

**Abstract:**

**Background:**

Despite growing interest in the therapeutic potential of 3,4-methylenedioxymethamphetamine (MDMA), no targeted measure to systematically assess side effects of MDMA-assisted psychotherapy (MDMA-AP) exists.

**Objective:**

Our aim was to develop an MDMA-Assisted Psychotherapy Side Effects Tool (M-SET) to capture side effects over the course of MDMA-AP.

**Methods:**

Informed by a systematic review and a review of other relevant questionnaires, we drafted a list of potential side effects. Face and content validation were obtained via a modified two-round online Delphi process involving experts in MDMA-AP and the neuropsychopharmacology of MDMA.

**Findings:**

Twelve experts consented to participate over two rounds of Delphi panel deliberations (response rate: Round 1 = 83–92%, Round 2 = 75%). The Delphi panellists were asked to keep, discard, modify or suggest additional items. The final version of the M-SET consists of 165 items across four questionnaires that collect information at screening, baseline, the day of medication sessions and longer term follow-up.

**Conclusions:**

The use of a modified Delphi technique proved a successful method to generate content for the first structured tool designed to evaluate side effects specifically associated with MDMA-AP.

**Clinical implications:**

The M-SET is recommended for use in both research and clinical settings. Its implementation has the potential to improve the safety of delivering MDMA-AP as well as support the development of a more systematic and robust evidence base on its safety and tolerability.

**STRENGTHS AND LIMITATIONS OF THIS STUDY**

- The MDMA-Assisted Psychotherapy Side Effects Tool (M-SET) was developed using a robust Delphi technique with a diverse panel of experts in the field.
- The M-SET can be used in both research and clinical settings to systematically assess side effects and guide clinical decision-making during MDMA-assisted psychotherapy.
- A small number of items (>14%) did not meet consensus after two Delphi rounds, and non-consensus was resolved by the research team, potentially introducing bias. Delphi panellists reviewed and endorsed the final version of the M-SET to reduce any bias.
- The M-SET comprises a large number of items (including 49 during acute drug administration) which may impose a burden on participants; subsequent iterations should aim to shorten the instrument.
- The M-SET will need to undergo reliability, validity and sensitivity testing and refinement over time.

## Background

 In response to the need for new approaches to the treatment of mental disorders, there has been a recent resurgence of investigation into the therapeutic use of 3,4-methylenedioxymethamphetamine (MDMA) combined with psychotherapy, for example, see ref 1. Based on promising results from clinical trials for post-traumatic stress disorder (PTSD),^1 2^ in 2023 the Australian Therapeutic Goods Administration approved the prescription of MDMA by authorised psychiatrists for this indication.^3^ Limited clinical use of MDMA-assisted psychotherapy (MDMA-AP) is also approved in Canada and Switzerland. While the first New Drug Application for MDMA-AP for PTSD to the US Food and Drug Administration was unsuccessful due to limitations of existing data, clinical research in this area is ongoing.^4^

While trials to date have largely concluded that MDMA-AP is safe and well tolerated, concerns have been raised about unique risks associated with this and similar treatment approaches. In particular, attention has been drawn to risks arising from combining psychotherapy with substances that may increase suggestibility and impair capacity for informed consent.^5 6^ One putative mechanism of MDMA-AP is that MDMA facilitates the emergence of challenging emotions and memories, which can then be processed with the guidance of a clinician.^7^ However, if negative material is not managed with an appropriately skilled clinician, MDMA-AP could result in unintended harms to vulnerable individuals.^5^ Such harms are not yet well understood or adequately measured and reported in existing research protocols.^5 8^

In a recent systematic review and meta-analysis examining the side effects of MDMA-AP across psychiatric indications, we found that undergoing MDMA-AP increased the likelihood of experiencing acute short-term and residual side effects compared with placebo mainly during the treatment days.^8^ However, we also found that most studies did not systematically assess the safety and tolerability of MDMA-AP; clinical trials in patients to date have primarily relied on spontaneous reporting of side effects, with limited use of structured assessment tools to capture safety data outside of suicidality and physiological (eg, blood pressure) measurement. Although this is standard practice in drug trials, there remains debate about the most appropriate method for collecting side effect data from participants.^9^ Evidence suggests that studies that rely on asking general questions about safety and tolerability, as opposed to using structured instruments, are at risk of underestimating the extent of adverse effects.^9^ To date, systematic measurement and reporting of side effects in trials of MDMA-AP have been hampered by the lack of structured measures designed to assess these side effects. This is of concern in terms of accurate characterisation of the risk-benefit profile of MDMA-AP. It is also relevant in the context of dose titration decisions (ie, whether to administer a supplemental dose within a session or increase dose between sessions), which require information about how well participants tolerate these doses. In addition, evidence suggests that MDMA-AP may have a unique side effect profile relative to other treatments, including mystical experiences, possible increased attachment to therapists and changes in personality.^6 8 10^ While other scales such as the Systematic Assessment for Treatment Emergent Events or psychiatric side effect scales have the capacity to assess general side effects, a more specific tool is needed to robustly measure and characterise MDMA-AP side effects during and after the course of treatment.

## Objectives

To address this issue—and based on the approach taken in the development of the Ketamine Side Effects Tool (KSET),^11^ we developed an MDMA-Assisted Psychotherapy Side Effects Tool (M-SET). The M-SET systematically captures safety information across four time periods: screening (for potential contraindications); baseline (prior to commencement of MDMA-AP); acute treatment (the day of medication sessions); and follow-up (in the weeks to months following a course of treatment). The tool aims to: (1) ensure potential contraindications are comprehensively assessed prior to enrolment in MDMA-AP; (2) inform dosing decisions during and between MDMA-AP sessions; and (3) encourage greater consistency in the measurement of side effects and the development of a more robust evidence on the safety and tolerability of MDMA-AP. The tool further aims to have utility in both clinical trials and for use in the clinical implementation of MDMA-AP.

## Methods

Development of the M-SET included four phases: (1) a systematic review and meta-analysis of side effects associated with MDMA-AP that has been published separately^8^; (2) review of items from other relevant measures of mental disorder symptoms, drug effects and altered states of consciousness; (3) development of a draft M-SET based on the results of phase I and phase II; and (4) face and content validation and revision of the draft M-SET based on feedback from experts via a Delphi technique.^12^ This Delphi process adhered to the Guidance on Conducting and REporting DElphi Studies (CREDES) checklist (online supplemental table S1).^13^

### Preparation for Round 1

The initial pool of items for the M-SET was generated by extracting the side effects reported by participants who underwent MDMA-AP in studies identified through a systematic review and meta-analysis available separately.^8^ To ensure that a comprehensive pool of possible side effects was considered during the Delphi process and to mitigate potential under-reporting of side effects in existing MDMA-AP clinical trials due to the use of spontaneous reports, these were supplemented by items from other relevant measures, including the KSET,^11^ Brief Psychiatric Rating Scale,^14^ Clinician-Administered Dissociative States Scale,^15^ Drug Effects Questionnaire^16^ and Altered States of Consciousness Rating Scale.^17 18^ Additional items were also drawn from an inventory of unusual experiences among meditators^19^ on the basis that meditation is thought to induce non-ordinary states of consciousness in a manner that may be similar to the effects of psychedelic substances.^20^ The initial pool of items by source (ie, from the systematic review, existing questionnaires or other research) is included in online supplemental table S2.

Based on the above process, a draft M-SET was developed, and formal feedback from experts regarding its face and content validity was then obtained via the Delphi technique—an established method for developing the content of questionnaires.^21^ The Delphi technique seeks to obtain consensus on experts’ opinions, termed panel members, through a series of structured surveys. As part of the process, the responses from each round are fed back in summarised form to the panel members who are then given an opportunity to respond again to the emerging data, with a view to gaining consensus rather than agreement from the panellists.^21^ To minimise unnecessary time imposed on panellists and to maximise engagement, we used a modified two-round online Delphi process,^21 22^ in which an item pool was generated prior to the panellist consideration in Round 1.

### Panel selection

Panel members included researchers and clinicians with significant involvement in research investigating the therapeutic use in clinical populations or acute administration of MDMA in healthy volunteers. Panel members were identified based on third-party recommendations by the research team’s networks and a review of the MDMA-AP literature and of human laboratory studies investigating the acute effects of MDMA. Potential panellists were searched on PubMed and selected if they had senior or first authorship on ≥1 published empirical study investigating MDMA-AP or >5 publications on the acute effects of MDMA in humans, and prioritised based on years of experience and quantity of publications. Given the limited literature on MDMA-AP to date, one publication was deemed sufficient for inclusion as a panel member with expertise in this area.

To obtain a wide range of perspectives, 24 researchers and clinicians were approached from >10 international institutions. We aimed to recruit at least 5–10 panellists necessary to obtain adequate content validation.^23^ Potential panel members were approached via email by the lead investigator, who outlined the goals and processes of the project. Written informed consent was obtained from every participant. All procedures contributing to this work comply with the ethical standards of the relevant national and institutional committees on human experimentation and with the Helsinki Declaration of 1975, as revised in 2008 and 2024.

### Round 1

Panel members completed an online questionnaire in which they voted to ‘keep’, ‘discard’ or ‘modify’ each of the items in the draft M-SET. Panel members had the opportunity to provide open comments on the tool’s overall practicality and functionality and suggest modifications. Experts were allowed approximately 1 month to provide their first round of feedback. Panellists did not see the other panel members’ comments until Round 1 was completed.

80% is an appropriate cut-off for achieving content validity when at least 10 experts participate in the consensus process.^23^ Therefore, if 80% of panel members voted to keep an item, it was accepted in the final M-SET, while if 80% of panel members voted to discard an item, it was omitted. Items not meeting the 80% threshold in Round 1 were modified according to feedback and redistributed to the panellists for Round 2. Qualitative feedback was collated and reviewed independently by one member of the research team (JC), who identified where multiple experts made similar comments. The results of the preliminary review were then presented to project supervisors (AAG, GB) to discuss whether each aspect of the M-SET should be retained, revised or removed. Feedback was automatically endorsed if ≥3 experts commented unanimously on the same issue. Decisions for all other comments and feedback were made based on discussion and consensus within the research team. A report was compiled and sent to panel members, which included the results and responses from Round 1, with corresponding feedback from the research team. Results were deidentified to reduce the risk of bias and influence.

### Round 2

In Round 2, experts were asked to provide feedback using the same method as described in Round 1, but with the knowledge of the group scores and comments. Experts were allowed approximately 1 month to provide their second round of feedback. The review and revision process described in Round 1 was repeated by the research team. Items that did not reach consensus in round 2 but reached an 80% threshold when keep and modify responses were combined were reviewed on a case-by-case basis by the core research team (JC, AAG and GB), who assessed whether to include or discard these items via consensus. To reduce bias, the final tool was approved and endorsed by all panellists prior to submission for publication. All other items that did not reach consensus were omitted from the final M-SET.

### Patient and public involvement

Patients and/or the public were not involved in the design, or conduct, or reporting, or dissemination plans of this research.

## Findings

### Panellists and response rates

Twenty-four potential panel members were invited, of whom 12 consented to participate in the Delphi process. Six were involved in research investigating the safety and efficacy of MDMA-AP, while the other six had expertise in the neuropsychopharmacology of MDMA. The panel included members from the USA (58%), Switzerland (25%) and the UK (17%). The response rate for each item was 83–92% and 75% for Round 1 and Round 2, respectively. While a reduction in the response rate occurred across the two rounds, the overall response rate was satisfactory, given that a minimum response of 70% is thought to maintain the rigour of the Delphi technique.^24^

### Round 1 results

**Figure 1:**
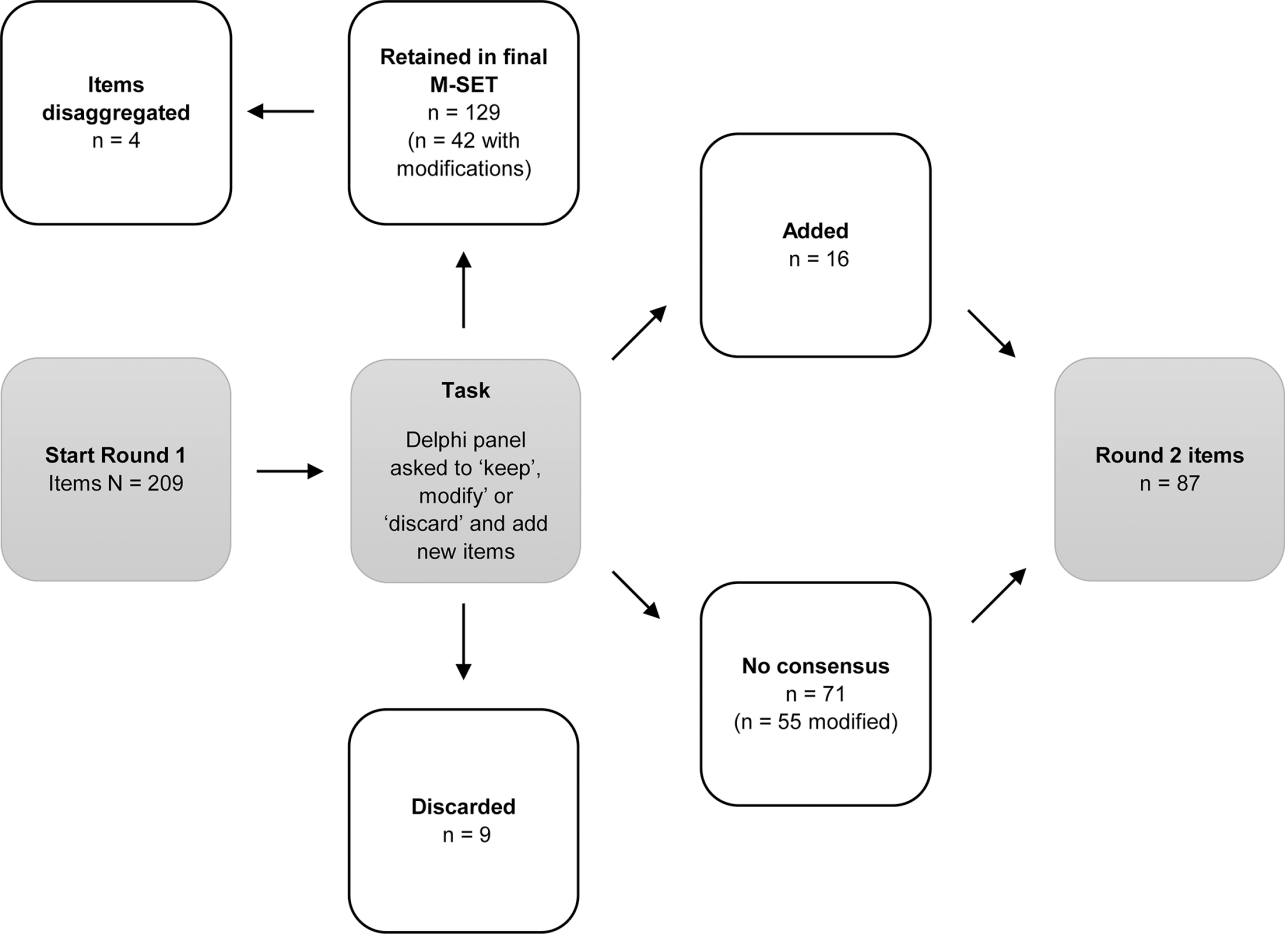
Results for round 1. M-SET, MDMA-Assisted Psychotherapy Side Effects Tool.

Of the 209 items compiled before Round 1 deliberations, 129 reached consensus for inclusion in the final M-SET, nine reached consensus to be discarded and 71 did not reach consensus, so they were retained for consideration in Round 2 (figure 1). Of the 129 items that reached consensus for inclusion, three of these were disaggregated based on feedback, resulting in an additional four items included in the final M-SET after Round 1. For example, ‘drowsiness, fatigue and/or weakness’ was disaggregated into three separate items: ‘drowsiness’, ‘fatigue’ and ‘feeling weak’. Sixteen items were also added to the item pool in response to suggestions by the panel. When combined with the items that did not reach consensus in Round 1 (n=71), this resulted in a total of 87 items for Round 2. In addition, 97 items were modified according to feedback, of which 42 were included in the final M-SET and 55 were considered in Round 2. All changes made to items from Round 1 are provided in online supplemental table S3.

### Round 2 results

**Figure 2:**
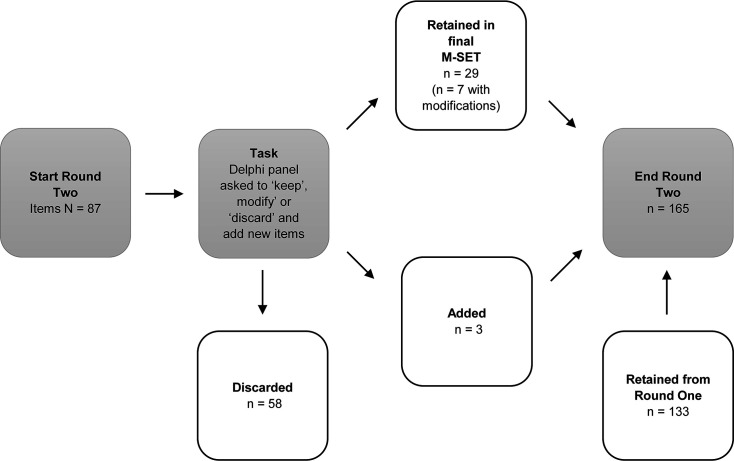
Results for round 2. M-SET, MDMA-Assisted Psychotherapy Side Effects Tool.

Of the 87 items at the commencement of Round 2 deliberations, 29 were included in the final M-SET (of which seven were modified according to feedback), and 58 were discarded (figure 2). Of the 29 items that were included in the final M-SET, 12 reached consensus when ≥80% of panel members voted to keep the item, while the remaining 17 items reached the 80% threshold when keep and modify responses were combined and were retained based on the research team’s judgement. Three items were added in response to suggestions by the panel. When combined with the items that reached consensus for inclusion in Round 1 (n=133), this resulted in a total of 165 items at the end of Round 2. All changes made to items from Round 2 are provided in online supplemental table S4.

### M-SET: final version

The final version of the M-SET is designed to systematically assess side effect information across four time periods: screening, baseline, acute treatment, and follow-up (online supplemental appendix 1). The tool is intended for use in both clinical and research settings and assesses (1) relevant history and comorbidities at screening; (2) baseline symptoms; (3) acute side effects during medication sessions to guide dose titration decisions within and between sessions; and (4) side effects experienced after the treatment period at follow-up. In addition to the side effects identified during the Delphi process, the acute treatment questionnaire also includes sections to monitor vital signs during medication sessions; record dosing details (eg, time of administration, dose in milligram); obtain tolerability ratings (made by a clinician); and complete a discharge assessment. Domains covered by each module of the M-SET are summarised in table 1.

**Table 1:** MDMA-Assisted Psychotherapy Side Effects Tool (M-SET) modules and domains assessed

Module	Relevant medical history	Relevant psychiatric history	Physical examination (eg, weight, height)	Clinical investigations (eg, blood, ECG)	Physical and physiological symptoms	Psychological symptoms	Vital signs	Acute drug effect
Screening questionnaire	✔	✔	✔	✔	✔	✔	✔	
Baseline questionnaire					✔	✔		
Acute questionnaire					✔	✔	✔	✔
Follow-up questionnaire					✔	✔	✔	

## Discussion

The M-SET aims to comprehensively cover potential side effects of MDMA-AP over an entire course of treatment, as well as follow-up, which has not been adequately assessed in the literature to date.^8^ The tool also facilitates screening of pre-existing conditions that may increase the risk of administering MDMA-AP, noting that specific contraindications or exclusion criteria will vary depending on the clinical or research protocol being used. Collecting this information in one standardised tool has the potential to improve the safety of implementing MDMA-AP, as well as support the development of a more robust evidence base on its safety and tolerability. In Australia and other countries which are expected to approve MDMA-AP for the treatment of PTSD or in Switzerland where MDMA-AP is established within the Limited Use Program,^25 26^ we anticipate that the use of the M-SET may encourage standardisation of real-world evidence on the safety of MDMA-AP in clinical practice.

While emerging literature on the efficacy of MDMA-AP shows great promise, side effects are likely under-reported due to a lack of systematic assessment.^8^ Although there is no clear consensus on the most appropriate method for assessing side effects from participants in drug trials, it is well established that studies that rely on general inquiry or spontaneous reporting are at risk of underdetecting side effects when compared with those using more systematic methods (eg, checklists or rating scales).^9^ In this context, a more structured assessment of side effects should be implemented when less is known about the safety profile of a drug.^9^ Evidence on MDMA-AP safety consists of a limited number of studies with highly selective samples.^8^ For example, in the initial phase III clinical trial investigating MDMA-AP, less than 7% of the people who were initially screened received treatment.^2^ While this is appropriate for this early stage of research, side effects will likely be greater when MDMA-AP is implemented beyond controlled clinical trials in patients with comorbidities or complex needs.^27^ Systematic assessment and monitoring of side effects will therefore be particularly pertinent when implementing MDMA-AP in broader populations where the risk-benefit profile of MDMA-AP is not yet well understood. Moreover, we think an approach favouring high sensitivity represents good research and clinical practice at this stage of the evidence. It is anticipated that implementation of the M-SET will improve the detection of rare and other clinically significant side effects.

### Limitations and recommendations

Because the initial item pool largely drew from a systematic review of the literature on MDMA-AP,^8^ we cannot rule out the presence of publication bias. Attempts were made to minimise this by drawing additional items from other measures. In addition, after the two Delphi rounds, there was still a large degree of non-consensus, with just under 14% of the Round 2 items reaching 80% agreement when keep, discard and modify responses were analysed separately. To resolve non-consensus, the research team had to exercise judgements about whether to include or exclude certain items from the M-SET, which had the potential to introduce researcher bias into the Delphi process. While it is possible that a further round of feedback may have increased the degree of consensus, we could not be confident that an adequate response rate would have been achieved,^24^ particularly given there was a drop in the response rate of 8–17% between Round 1 and Round 2. Lack of consensus or stable disagreement may reflect the early stages of this research and the diverse background of researchers involved; it is perhaps unsurprising that there are different perspectives about likely side effects of MDMA-AP given the evidence regarding this topic is still emerging. In this context, the research team erred on the side of ensuring the tool comprehensively captures possible side effects, as we move towards greater consensus on the side effect profile of MDMA-AP. One potential limitation of this approach is that more comprehensive assessments are time-consuming and may therefore lack clinical utility. For example, the M-SET acute questionnaire assesses 49 different symptoms at multiple time points on the day of medication sessions. This has the potential to impose a burden on participants. However, this is somewhat mitigated by the fact that some items use clinician ratings and observation rather than participant self-report, and some of the self-reported sections can be completed using digital technologies (eg, tablets or mobile phones) to reduce participant burden. In addition, researchers or clinicians can adapt the timing and specific content of the questionnaire to suit their particular contexts. To further reduce burden on participants and patients, it is important to explore the development of shortened versions of the M-SET for routine clinical use. Lastly, while we conducted a robust Delphi process with an expert panel of researchers and clinicians with significant involvement in administration of MDMA to humans and delivery of MDMA-AP, future research should aim to refine the M-SET with consultation from patient advocates, regulators and other relevant stakeholder groups. Taken together, it is recommended that the M-SET be tested and validated in healthy and clinical populations and refined over time. This should include an evaluation of the tool’s utility, including the practicalities of its current length and design in a range of populations, including people with comorbidities as new side effects may emerge.

### Clinical implications

Systematic assessment of side effects of MDMA-AP is recommended for any clinical trials into the safety and efficacy of MDMA-AP as well as during clinical implementation. The use of a modified Delphi technique proved a successful method to generate content for the first structured tool designed to evaluate side effects specifically associated with MDMA-AP. The M-SET is recommended for use in both research and clinical settings. While testing of the tool’s utility, validity and reliability is required, its implementation has the potential to improve the safety of delivering MDMA-AP, as well as support the development of a more robust evidence base on safety and tolerability over time.

## Supplementary material

10.1136/bmjopen-2025-105630 online supplemental file 1

10.1136/bmjopen-2025-105630 online supplemental file 2

## Data Availability

Data are available upon reasonable request.
