# Supplementary material for: Development of the MDMA-Assisted Psychotherapy Side Effects Tool (M-SET): a Delphi study
Source: BMJ Open. 2026 May 11;16(5):e105630. doi: 10.1136/bmjopen-2025-105630 (PMC13182389; doi:10.1136/bmjopen-2025-105630)
Supplement: online supplemental file 1 [file bmjopen-16-5-s001.pdf]

M-SET – Screening

Date of Assessment	__ / ___ / __	Participant Initials	__ __ __	Participant ID	

Instructions: The MSET Screening Questionnaire is a clinician-administered tool designed to be administered by a medical doctor.

The questionnaire is intended to guide the collection of relevant information during screening. Specific contraindications or exclusion criteria will vary, depending on the clinical or research protocol being used.

Please complete when assessing patient suitability for MDMA-assisted psychotherapy, providing further details in the **Clinical Notes** if required.

Relevant History and Comorbidities

1. Current or lifetime psychotic disorder (e.g., schizophrenia)	☐0 No	☐1 Yes
2. Current or lifetime bipolar disorder	☐0 No	☐1 Yes
3. Current or lifetime borderline personality disorder	☐0 No	☐1 Yes
4. Current or lifetime panic attacks	☐0 No	☐1 Yes
5. Current uncontrolled hypertension	☐0 No	☐1 Yes
6. Current or lifetime heart/cardiovascular condition<br>If yes, please specify: ______________________________	☐0 No	☐1 Yes
7. Current or lifetime cerebrovascular condition, including previous stroke<br>If yes, please specify: ______________________________	☐0 No	☐1 Yes
8. Current or lifetime liver condition<br>If yes, please specify: ______________________________	☐0 No	☐1 Yes
9. Current or lifetime kidney condition<br>If yes, please specify: ______________________________	☐0 No	☐1 Yes
10. Current or lifetime autoimmune condition (e.g., fibromyalgia, lupus, multiple sclerosis)<br>If yes, please specify: ______________________________	☐0 No	☐1 Yes
11. Infection with significant impact on physical health within past 4 weeks<br>If yes, please specify: ______________________________	☐0 No	☐1 Yes
12. Current or lifetime seizures, including epilepsy<br>If yes, controlled or uncontrolled?: ______________________	☐0 No	☐1 Yes
13. Current or lifetime glaucoma<br>If yes, controlled or uncontrolled?: ______________________	☐0 No	☐1 Yes
14. Alcohol or drug use during the past 6 months<br>If yes, please specify:<br>Frequency: __________<br>Quantity: __________<br>**Note:** Further assessment for current Substance Use Disorder, including physiological dependence and capacity to comply with the treatment protocol may be required.	☐0 No	☐1 Yes
15. Current pregnancy or plans to become pregnant<br>Mode of contraception, if any: ______________________	☐0 No	☐1 Yes
16. Current breastfeeding	☐0 No	☐1 Yes
17. Allergies (food and/or drug)<br>If yes, please list: ______________________	☐0 No	☐1 Yes

M-SET – Screening

Date of Assessment	_ _ / _ _ _ / _ _	Participant Initials	___ ___ ___	Participant ID	

History of MDMA Use

Have you ever received or used MDMA? (Check all that apply)

☐$_0$ No previous use
☐$_1$ Psychiatric treatment, with professional support → If yes, was it helpful? ☐$_0$ No ☐$_1$ Yes
☐$_2$ Therapeutic use in non-medical/non-research setting → If yes, was it helpful? ☐$_0$ No ☐$_1$ Yes
☐$_3$ Recreational use (e.g., Ecstasy, Molly)
☐$_4$ Other → Please specify ______________________

	Psychiatric treatment	Therapeutic use in non-medical/non-research setting	Recreational use	Other
When did you last take MDMA, Ecstasy or Molly?	☐$_1$ Last 3 months<br>☐$_2$ 3-12 months ago<br>☐$_3$ >12 months ago	☐$_1$ Last 3 months<br>☐$_2$ 3-12 months ago<br>☐$_3$ >12 months ago	☐$_1$ Last 3 months<br>☐$_2$ 3-12 months ago<br>☐$_3$ >12 months ago	☐$_1$ Last 3 months<br>☐$_2$ 3-12 months ago<br>☐$_3$ >12 months ago
How many occasions in the last year?				
How many occasions in total (lifetime)?				
How did you take it?	☐$_1$ By mouth<br>☐$_2$ Intranasally / 'snorting'<br>☐$_3$ IV<br>☐$_4$ Suppository<br>☐$_5$ Other, specify:<br>__________	☐$_1$ By mouth<br>☐$_2$ Intranasally / 'snorting'<br>☐$_3$ IV<br>☐$_4$ Suppository<br>☐$_5$ Other, specify:<br>__________	☐$_1$ By mouth<br>☐$_2$ Intranasally / 'snorting'<br>☐$_3$ IV<br>☐$_4$ Suppository<br>☐$_5$ Other, specify:<br>__________	☐$_1$ By mouth<br>☐$_2$ Intranasally / 'snorting'<br>☐$_3$ IV<br>☐$_4$ Suppository<br>☐$_5$ Other, specify:<br>__________
In what form?	☐$_1$ Crystals<br>☐$_2$ Pills<br>☐$_3$ Other, specify:<br>__________	☐$_1$ Crystals<br>☐$_2$ Pills<br>☐$_3$ Other, specify:<br>__________	☐$_1$ Crystals<br>☐$_2$ Pills<br>☐$_3$ Other, specify:<br>__________	☐$_1$ Crystals<br>☐$_2$ Pills<br>☐$_3$ Other, specify:<br>__________
Highest dose you think you've received/taken at one time	☐$_1$ <100mg<br>☐$_2$ 100-200mg<br>☐$_3$ >200mg<br>☐$_4$ Don't know	☐$_1$ <100mg<br>☐$_2$ 100-200mg<br>☐$_3$ >200mg<br>☐$_4$ Don't know	☐$_1$ <100mg<br>☐$_2$ 100-200mg<br>☐$_3$ >200mg<br>☐$_4$ Don't know	☐$_1$ <100mg<br>☐$_2$ 100-200mg<br>☐$_3$ >200mg<br>☐$_4$ Don't know
How sure were you that what you took was actually MDMA? (1 to 10; 1 = not sure at all; 10 = very sure)				

Current/past 6 months use of medications or supplements?

☐$_1$ Yes	☐$_0$ No
If "Yes" list all below:	
1. ______________________	Dose: ________<br>Frequency of use: ________<br>Years on medication/supplement: ________
2. ______________________	Dose: ________<br>Frequency of use: ________<br>Years on medication/supplement: ________
3. ______________________	Dose: ________<br>Frequency of use: ________<br>Years on medication/supplement: ________
4. ______________________	Dose: ________<br>Frequency of use: ________<br>Years on medication/supplement: ________
5. ______________________	Dose: ________<br>Frequency of use: ________<br>Years on medication/supplement: ________

M-SET – Screening

Date of Assessment	__ / ___ / __	Participant Initials	__ __ __	Participant ID	

Physical Examination			
1. **Weight**	________kg	☐-9	Missing
2. **Height**	________cm	☐-9	Missing
3. **Blood pressure - systolic**	________mmHg	☐-9	Missing
4. **Blood pressure - diastolic**	________mmHg	☐-9	Missing
5. **Pulse**	________BPM	☐-9	Missing
6. **Respiration Rate**	________(breaths per minute)	☐-9	Missing

Clinical Investigations (at clinical discretion)		
Collection date	DD/MMM/YY	
**Liver Function Tests (e.g., ALT, AST, ALP, GGT)**		
☐0 Normal	☐1 Abnormal	☐-9 Missing
If abnormal, please list abnormal markers and values:		
**Electrocardiogram (ECG/EKG)**		
☐0 Normal	☐1 Abnormal	☐-9 Missing
If abnormal, please list abnormal markers and values:		
**Other, please specify: ___________**		
☐0 Normal	☐1 Abnormal	☐-9 Missing
If abnormal, please list abnormal markers and values:		

Clinical Notes

Unless otherwise indicated, this form was completed by:
Signature: ***Date:*** DD/MMM/YY

M-SET – Baseline

Date of Assessment	__ / ___ / __	Participant Initials	__ __ __	Participant ID	

Instructions: The MSET Baseline Questionnaire should be completed by a clinician or researcher before commencing MDMA-assisted psychotherapy treatment to establish a baseline for potential side effects of treatment.

Rate items 1 to 58 based on the patient's self-report, using the following prompt: "Have you experienced the following symptoms over the past week? If yes, how severe were they?"

Symptoms<br>Check relevant items, providing further details as necessary in the Clinical Notes (e.g., pre-existing conditions, treatments, etc.)	Not in the past week	Severity<br>Mild – transient and easily tolerated<br>Moderate – caused discomfort and/or interference with usual activities<br>Severe – caused significant discomfort and/or considerable interference with usual activities		
		Mild	Moderate	Severe
1. Anxiety	☐$_0$	☐$_1$	☐$_2$	☐$_3$
2. Fear of losing self-control	☐$_0$	☐$_1$	☐$_2$	☐$_3$
3. Panic attacks	☐$_0$	☐$_1$	☐$_2$	☐$_3$
4. Restlessness	☐$_0$	☐$_1$	☐$_2$	☐$_3$
5. Irritable mood	☐$_0$	☐$_1$	☐$_2$	☐$_3$
6. Low/depressed mood	☐$_0$	☐$_1$	☐$_2$	☐$_3$
7. Anguish or despair	☐$_0$	☐$_1$	☐$_2$	☐$_3$
8. Unprompted inconsolable crying	☐$_0$	☐$_1$	☐$_2$	☐$_3$
9. Rumination (i.e., repeated negative thoughts that are hard to control)	☐$_0$	☐$_1$	☐$_2$	☐$_3$
10. Suicidal thoughts	☐$_0$	☐$_1$	☐$_2$	☐$_3$
11. Suicidal behaviour (e.g., suicide attempt, interrupted or aborted attempt, preparatory acts)	☐$_0$	☐$_1$	☐$_2$	☐$_3$
12. Thoughts of self-harm and/or intentional self-harm, without suicidal intent	☐$_0$	☐$_1$	☐$_2$	☐$_3$
13. Repeated, disturbing memories, thoughts, or images of a stressful experience from the past	☐$_0$	☐$_1$	☐$_2$	☐$_3$
14. Feeling threatened	☐$_0$	☐$_1$	☐$_2$	☐$_3$
15. Feeling disconnected from friends, family, or social group	☐$_0$	☐$_1$	☐$_2$	☐$_3$
16. Feeling that it's hard to connect with others or that socializing is a real effort	☐$_0$	☐$_1$	☐$_2$	☐$_3$
17. Lack of drive or motivation to pursue goals previously valued as meaningful	☐$_0$	☐$_1$	☐$_2$	☐$_3$
18. Impaired ability to do normal work/study, potentially putting employment/education at risk	☐$_0$	☐$_1$	☐$_2$	☐$_3$
19. Difficulties with memory and/or concentration	☐$_0$	☐$_1$	☐$_2$	☐$_3$
20. Difficulty making even the smallest decisions	☐$_0$	☐$_1$	☐$_2$	☐$_3$
21. Thoughts and/or actions feeling slowed down or sped up	☐$_0$	☐$_1$	☐$_2$	☐$_3$
22. Experienced your surroundings as strange and/or weird	☐$_0$	☐$_1$	☐$_2$	☐$_3$
23. Things seeming to be unreal or dreamlike	☐$_0$	☐$_1$	☐$_2$	☐$_3$
24. Feeling separated from what is happening around you (e.g., as if you are in the movie or a play)	☐$_0$	☐$_1$	☐$_2$	☐$_3$
25. Feeling disconnected from your own body or looking at things from outside your body	☐$_0$	☐$_1$	☐$_2$	☐$_3$
26. Your sense of your own body changed (e.g., felt unusually large or small)	☐$_0$	☐$_1$	☐$_2$	☐$_3$
27. Objects looked different to what you would expect (e.g., distorted or unreal)	☐$_0$	☐$_1$	☐$_2$	☐$_3$
28. Seeing things as if you were in a tunnel, or looking through a wide-angle photographic lens	☐$_0$	☐$_1$	☐$_2$	☐$_3$
29. Things seemed to take much longer than you would have expected (e.g., as if time is passing slowly/standing still)	☐$_0$	☐$_1$	☐$_2$	☐$_3$
30. Things seemed to be happening very quickly, as if there is a lifetime in a moment	☐$_0$	☐$_1$	☐$_2$	☐$_3$
31. Sounds almost disappeared or became much stronger than you would have expected	☐$_0$	☐$_1$	☐$_2$	☐$_3$
32. Things seeming very real, as if there is a special sense of clarity	☐$_0$	☐$_1$	☐$_2$	☐$_3$
33. Unprompted hysterical laughter	☐$_0$	☐$_1$	☐$_2$	☐$_3$
34. Drowsiness	☐$_0$	☐$_1$	☐$_2$	☐$_3$
35. Fatigue	☐$_0$	☐$_1$	☐$_2$	☐$_3$
36. Feeling weak	☐$_0$	☐$_1$	☐$_2$	☐$_3$
37. Dizziness	☐$_0$	☐$_1$	☐$_2$	☐$_3$
38. Insomnia (e.g., difficulty falling asleep, staying asleep and/or other sleep problems)	☐$_0$	☐$_1$	☐$_2$	☐$_3$
39. Nightmares	☐$_0$	☐$_1$	☐$_2$	☐$_3$
40. Feeling the need for much less or much more sleep than usual	☐$_0$	☐$_1$	☐$_2$	☐$_3$
41. Diarrhoea	☐$_0$	☐$_1$	☐$_2$	☐$_3$
42. Nausea and/or vomiting	☐$_0$	☐$_1$	☐$_2$	☐$_3$
43. Lower than typical appetite	☐$_0$	☐$_1$	☐$_2$	☐$_3$
44. Lack of interest in sex	☐$_0$	☐$_1$	☐$_2$	☐$_3$

M-SET – Baseline

Date of Assessment	_ _ / _ _ _ / _ _	Participant Initials	__ __ __	Participant ID	

45. Physical pain<br>If yes, where?: ____________________	☐$_0$	☐$_1$	☐$_2$	☐$_3$
46. Headache or migraine	☐$_0$	☐$_1$	☐$_2$	☐$_3$
47. Muscle tension	☐$_0$	☐$_1$	☐$_2$	☐$_3$
48. Jaw clenching/tight jaw	☐$_0$	☐$_1$	☐$_2$	☐$_3$
49. Burning, prickling or tingling sensations	☐$_0$	☐$_1$	☐$_2$	☐$_3$
50. Somatic energy, vibrations, or currents through your body	☐$_0$	☐$_1$	☐$_2$	☐$_3$
51. Muscle twitching	☐$_0$	☐$_1$	☐$_2$	☐$_3$
52. Involuntary eye movements (e.g., eye wiggles)	☐$_0$	☐$_1$	☐$_2$	☐$_3$
53. Blurred vision	☐$_0$	☐$_1$	☐$_2$	☐$_3$
54. Sensitivity to cold/feeling cold	☐$_0$	☐$_1$	☐$_2$	☐$_3$
55. Impaired gait/balance (e.g., difficulty walking, unsteadiness while standing or walking)	☐$_0$	☐$_1$	☐$_2$	☐$_3$
56. Dry mouth	☐$_0$	☐$_1$	☐$_2$	☐$_3$
57. Excessive thirst	☐$_0$	☐$_1$	☐$_2$	☐$_3$
58. Frequent urination or urge to urinate	☐$_0$	☐$_1$	☐$_2$	☐$_3$

Clinical Notes

Unless otherwise indicated, this form was completed by:
Signature: ***Date:*** D D / M M M / Y Y

Instructions: Rate items 59 to 64 based on the patient's self-report over the past week and observed behaviour. Items 65 to 68 are rated on the basis of observed behaviour and speech.

**Symptoms**<br>Check relevant items, providing further details as necessary in the **Clinical Notes** (e.g., pre-existing conditions, treatments, etc.)	Not in the past week	Severity<br>**Mild** – transient and easily tolerated<br>**Moderate** – caused discomfort and/or interference with usual activities<br>**Severe** – caused significant discomfort and/or considerable interference with usual activities		
		Mild	Moderate	Severe
59. Unusually elevated mood (e.g., exaggerated feeling of well-being, cheerfulness, euphoria and optimism)	☐$_0$	☐$_1$	☐$_2$	☐$_3$
60. Grandiosity (e.g., feeling like you had special powers or abilities that others don't recognise, or that you might be somebody rich or famous?)*	☐$_0$	☐$_1$	☐$_2$	☐$_3$
61. Suspiciousness (e.g., belief that other persons have acted maliciously or with bad intent)*	☐$_0$	☐$_1$	☐$_2$	☐$_3$
62. Hallucinations (e.g., seeing, hearing, smelling or tasting things that are not present in reality)*	☐$_0$	☐$_1$	☐$_2$	☐$_3$
63. Unusual thought content (e.g., delusions, ideas of reference/persecution)*	☐$_0$	☐$_1$	☐$_2$	☐$_3$
64. Disorientation (e.g., does not comprehend situations or communications, confusion regarding person, place, or time)*	☐$_0$	☐$_1$	☐$_2$	☐$_3$
65. Conceptual disorganisation (e.g., degree to which speech is confused, disconnected, vague or disorganised)*	☐$_0$	☐$_1$	☐$_2$	☐$_3$
66. Tension (e.g., observable signs of physical tension, `nervousness' and/or agitation)	☐$_0$	☐$_1$	☐$_2$	☐$_3$
67. Motor hyperactivity (e.g., increase in energy level evidenced in more frequent movement and/or rapid speech)*	☐$_0$	☐$_1$	☐$_2$	☐$_3$
68. Abnormal mannerisms and/or posturing (e.g., grimacing, rocking, nodding, postures which are clearly uncomfortable or inappropriate)*	☐$_0$	☐$_1$	☐$_2$	☐$_3$
69. Other (please specify):	☐$_0$	☐$_1$	☐$_2$	☐$_3$

*Endorsing this item at baseline as Moderate to Severe should be considered cause for further investigation and potential exclusion

M-SET Acute Treatment – Pre-Treatment

Date of Assessment	__/___/__	Participant Initials	__ __ __	Participant ID	

Pre-Treatment: Symptoms Today
Instructions: The MSET Acute Questionnaire should be completed by a clinician or researcher during an MDMA-assisted psychotherapy session.

Rate items 1 to 40 based on the patient's self-report, using the following prompt: "Have you experienced any of the following symptoms today?
If yes, how severe were they?"

**Symptoms**<br>**Check relevant items and severity,** providing further details as necessary in the **Clinician Notes** (e.g., any other observations during treatment)	**Not at all**	**Severity**<br>**Mild** – transient and easily tolerated<br>**Moderate** – caused discomfort and/or interference with usual activities<br>**Severe** – caused significant discomfort and/or considerable interference with usual activities		
		**Mild**	**Moderate**	**Severe**
1. Anxiety	☐$_0$	☐$_1$	☐$_2$	☐$_3$
2. Fear of losing self-control	☐$_0$	☐$_1$	☐$_2$	☐$_3$
3. Panic attacks	☐$_0$	☐$_1$	☐$_2$	☐$_3$
4. Restlessness	☐$_0$	☐$_1$	☐$_2$	☐$_3$
5. Irritable mood	☐$_0$	☐$_1$	☐$_2$	☐$_3$
6. Low/depressed mood	☐$_0$	☐$_1$	☐$_2$	☐$_3$
7. Rumination (i.e., repeated negative thoughts that are hard to control)	☐$_0$	☐$_1$	☐$_2$	☐$_3$
8. Suicidal thoughts	☐$_0$	☐$_1$	☐$_2$	☐$_3$
9. Repeated, disturbing memories, thoughts, or images of a stressful experience from the past	☐$_0$	☐$_1$	☐$_2$	☐$_3$
10. Feeling threatened	☐$_0$	☐$_1$	☐$_2$	☐$_3$
11. Trouble concentrating	☐$_0$	☐$_1$	☐$_2$	☐$_3$
12. Thoughts and/or actions feeling slowed down or sped up	☐$_0$	☐$_1$	☐$_2$	☐$_3$
13. Experiencing your surroundings as strange and/or weird	☐$_0$	☐$_1$	☐$_2$	☐$_3$
14. Things seeming to be unreal or dreamlike	☐$_0$	☐$_1$	☐$_2$	☐$_3$
15. Your sense of your own body changed (e.g., felt unusually large or small)	☐$_0$	☐$_1$	☐$_2$	
16. Objects looking different to what you would expect (e.g., distorted or unreal)	☐$_0$	☐$_1$	☐$_2$	☐$_3$

17. Things seem to be taking much longer or much less time than expected (e.g., as if time is passing slowly/standing still or there is a lifetime in a moment)	☐$_0$	☐$_1$	☐$_2$	☐$_3$
18. Sounds almost disappearing or becoming much stronger than you would have expected	☐$_0$	☐$_1$	☐$_2$	☐$_3$
19. Things seeming very real, as if there is a special sense of clarity	☐$_0$	☐$_1$	☐$_2$	☐$_3$
20. Feeling numb	☐$_0$	☐$_1$	☐$_2$	☐$_3$
21. Fear that you might or have said too much (over-disclosure, sharing of information you would rather have kept private)	☐$_0$	☐$_1$	☐$_2$	☐$_3$
22. Drowsiness	☐$_0$	☐$_1$	☐$_2$	☐$_3$
23. Fatigue	☐$_0$	☐$_1$	☐$_2$	☐$_3$
24. Feeling weak	☐$_0$	☐$_1$	☐$_2$	☐$_3$
25. Dizziness	☐$_0$	☐$_1$	☐$_2$	☐$_3$
26. Nausea and/or vomiting	☐$_0$	☐$_1$	☐$_2$	☐$_3$
27. Lower that typical appetite	☐$_0$	☐$_1$	☐$_2$	☐$_3$
28. Headache or migraine	☐$_0$	☐$_1$	☐$_2$	☐$_3$
29. Muscle tension	☐$_0$	☐$_1$	☐$_2$	☐$_3$
30. Jaw clenching/tight jaw	☐$_0$	☐$_1$	☐$_2$	☐$_3$
31. Burning, prickling or tingling sensations	☐$_0$	☐$_1$	☐$_2$	☐$_3$
32. Somatic energy, vibrations, or currents through your body	☐$_0$	☐$_1$	☐$_2$	☐$_3$
33. Muscle twitching	☐$_0$	☐$_1$	☐$_2$	☐$_3$
34. Involuntary eye movements (e.g., eye wiggles)	☐$_0$	☐$_1$	☐$_2$	☐$_3$
35. Blurred vision	☐$_0$	☐$_1$	☐$_2$	☐$_3$
36. Sensitivity to cold/feeling cold	☐$_0$	☐$_1$	☐$_2$	☐$_3$
37. Impaired gait/balance (e.g., difficulty walking, unsteadiness while standing or walking)	☐$_0$	☐$_1$	☐$_2$	☐$_3$
38. Dry mouth	☐$_0$	☐$_1$	☐$_2$	☐$_3$
39. Excessive thirst	☐$_0$	☐$_1$	☐$_2$	☐$_3$
40. Frequent urination or urge to urinate	☐$_0$	☐$_1$	☐$_2$	☐$_3$

M-SET Acute Treatment – Pre-Treatment

Date of Assessment	__ / ___ / __	Participant Initials	__ __ __	Participant ID	

Instructions: Rate items 42 to 47 based on the individual's self-report and observed behaviour since the medication was administered. Items 48 to 50 are rated on the basis of observed behaviour and speech.

**Symptoms**<br>Tick relevant items and provide further details in the **Clinical Notes** (e.g., pre-existing conditions, treatments, etc.)	**Not At All**	**Severity**<br>**Mild –** transient and easily tolerated<br>**Moderate –** caused discomfort and/or interference with usual activities<br>**Severe –** caused significant discomfort and/or considerable interference with usual activities		
		**Mild**	**Moderate**	**Severe**
42. Hostility (e.g., argumentative, aggressive, angry)	☐ 0	☐ 1	☐ 2	☐ 3
43. Grandiosity (e.g., feeling like you had special powers or abilities that others don't recognise, or that you might be somebody rich or famous?)	☐ 0	☐ 1	☐ 2	☐ 3
44. Suspiciousness (e.g., belief that other persons have acted maliciously or with bad intent)	☐ 0	☐ 1	☐ 2	☐ 3
45. Hallucinations (e.g., seeing, hearing, smelling or tasting things that are not present in reality)	☐ 0	☐ 1	☐ 2	☐ 3
46. Unusual thought content (e.g., delusions, ideas of reference/persecution)	☐ 0	☐ 1	☐ 2	☐ 3
47. Disorientation (e.g., does not comprehend situations or communications, confusion regarding person, place, or time)	☐ 0	☐ 1	☐ 2	☐ 3
48. Conceptual disorganisation (e.g., degree to which speech is confused, disconnected, vague or disorganised)	☐ 0	☐ 1	☐ 2	☐ 3
49. Tension (e.g., observable signs of physical tension, `nervousness' and agitation)	☐ 0	☐ 1	☐ 2	☐ 3
50. Motor hyperactivity (e.g., increase in energy level evidenced in more frequent movement and/or rapid speech)	☐ 0	☐ 1	☐ 2	☐ 3
51. Other (please specify):	☐ 0	☐ 1	☐ 2	☐ 3

Drug Effects Questionnaire

Instructions: This questionnaire asks about how you are feeling after taking the substance that was given to you. Please draw a mark on the line to show how strongly you are feeling each of the following effects <u>right now</u>. You can mark anywhere on the line, but please draw a vertical line (one that goes straight up and down).

Let's look at an example first.

Example: Do you feel dizzy right now?

If you do not feel dizzy, draw a line at NOT AT ALL. If you feel very dizzy, draw a line at EXTREMELY. If you feel somewhere in between, you can draw a mark anywhere along the line between NOT AT ALL and EXTREMELY to indicate how dizzy you are. For example, if you feel a little dizzy, you might draw a line that looks like the example below.

NOT AT ALL |—|———————————————| EXTREMELY

Note: If you have not yet taken any medication, please respond NOT AT ALL to the questions below

1. Do you **FEEL** a drug effect right now?

   NOT AT ALL |————————————————| EXTREMELY

2. Are you **HIGH** right now?

   NOT AT ALL |————————————————| EXTREMELY

3. Do you **DISLIKE** any of the effects you are feeling right now?

   NOT AT ALL |————————————————| EXTREMELY

4. Do you **LIKE** any of the effects you are feeling right now?

   NOT AT ALL |————————————————| EXTREMELY

5. Would you like **MORE** of the drug you took, right now?

   NOT AT ALL |————————————————| EXTREMELY

M-SET Acute Treatment – Pre-Treatment

Date of Assessment	__ / ___ / __	Participant Initials	__ __ __	Participant ID	

Pre-Treatment Physiological Measures

Time	__ : __		
1. **Blood pressure - systolic**	________mmHg	☐-9	Missing
2. **Blood pressure - diastolic**	________mmHg	☐-9	Missing
3. **Pulse**	________BPM	☐-9	Missing
4. **Temperature**	________°C.	☐-9	Missing

Pre-Treatment Orientation
Instructions: Record answer if incorrect

Time	__ : __		
	Incorrect	Correct	Answer if incorrect
1. Name	☐0	☐1	
2. Date of birth	☐0	☐1	
3. Age	☐0	☐1	
4. Year	☐0	☐1	
5. Month	☐0	☐1	
6. Date	☐0	☐1	
7. Day	☐0	☐1	
8. Place	☐0	☐1	
9. Suburb	☐0	☐1	
Score	__ / 9		

Treatment Administration Details

1. **Time dosing**	__ : __.
2. **Dose in mg**	________mg
3. **If dose was reduced (or not increased as indicated by protocol), please provide the reason**	☐-3 n/a<br>☐1 Could not tolerate last dose<br>☐2 Other. Please specify: ____________________

Approved for dosing:
Signature: *Date:* D D / M M / Y Y

Clinical Notes

Unless otherwise indicated, this form was completed by:
Signature: *Date:* D D / M M M / Y Y

M-SET Acute Treatment – X minutes Post-Treatment

Date of Assessment	__ / ___ / __	Participant Initials	___ ___	Participant ID	

Instructions: The MSET Acute Questionnaire should be completed by a study clinician during an MDMA-assisted psychotherapy session.

Rate items 1 to 41 based on the individual's self-report.

Questions for participant/patient:
"Have you experienced any of the following symptoms today, since the medication was administered?
If yes, how severe were they?"

Symptoms<br>**Tick relevant items and severity.** Further details can be documented by the clinician in **Clinician Notes**<br>(e.g., any other observations during treatment)	Not at all	Severity<br>**Mild** – transient and easily tolerated<br>**Moderate** – caused discomfort and/or interference with usual activities<br>**Severe** – caused significant discomfort and/or considerable interference with usual activities		
		Mild	Moderate	Severe
1. Anxiety	☐$_0$	☐$_1$	☐$_2$	☐$_3$
2. Fear of losing self-control	☐$_0$	☐$_1$	☐$_2$	☐$_3$
3. Panic attacks	☐$_0$	☐$_1$	☐$_2$	☐$_3$
4. Restlessness	☐$_0$	☐$_1$	☐$_2$	☐$_3$
5. Irritable mood	☐$_0$	☐$_1$	☐$_2$	☐$_3$
6. Low/depressed mood	☐$_0$	☐$_1$	☐$_2$	☐$_3$
7. Rumination (e.g., repeated negative thoughts that are hard to control)	☐$_0$	☐$_1$	☐$_2$	☐$_3$
8. Suicidal thoughts	☐$_0$	☐$_1$	☐$_2$	☐$_3$
9. Repeated, disturbing memories, thoughts, or images of a stressful experience from the past	☐$_0$	☐$_1$	☐$_2$	☐$_3$
10. Feeling threatened	☐$_0$	☐$_1$	☐$_2$	☐$_3$
11. Trouble concentrating	☐$_0$	☐$_1$	☐$_2$	☐$_3$
12. Thoughts and/or actions being slowed down or sped up	☐$_0$	☐$_1$	☐$_2$	☐$_3$
13. Experiencing your surroundings as strange and weird	☐$_0$	☐$_1$	☐$_2$	☐$_3$
14. Things seeming unreal or dreamlike	☐$_0$	☐$_1$	☐$_2$	☐$_3$
15. Your sense of your own body changed (e.g., felt unusually large or small)	☐$_0$	☐$_1$	☐$_2$	☐$_3$
16. Objects looking different to what you would expect (e.g., distorted, unreal)	☐$_0$	☐$_1$	☐$_2$	☐$_3$
17. Things seeming to take much longer than you would have expected (e.g., as if time is passing slowly/standing still)	☐$_0$	☐$_1$	☐$_2$	☐$_3$
18. Things seeming to happen very quickly, as if there is a lifetime in a moment	☐$_0$	☐$_1$	☐$_2$	☐$_3$
19. Sounds almost disappearing or becoming much stronger than you would have expected	☐$_0$	☐$_1$	☐$_2$	☐$_3$
20. Things seeming very real, as if there is a special sense of clarity	☐$_0$	☐$_1$	☐$_2$	☐$_3$
21. Feeling numb	☐$_0$	☐$_1$	☐$_2$	☐$_3$
22. Fear that you might or have said too much (over-disclosure, sharing of information you would rather have kept private)	☐$_0$	☐$_1$	☐$_2$	☐$_3$
23. Drowsiness	☐$_0$	☐$_1$	☐$_2$	☐$_3$
24. Fatigue	☐$_0$	☐$_1$	☐$_2$	☐$_3$
25. Feeling weak	☐$_0$	☐$_1$	☐$_2$	☐$_3$
26. Dizziness	☐$_0$	☐$_1$	☐$_2$	☐$_3$
27. Nausea and/or vomiting	☐$_0$	☐$_1$	☐$_2$	☐$_3$
28. Lower that typical appetite	☐$_0$	☐$_1$	☐$_2$	☐$_3$
29. Headache or migraine	☐$_0$	☐$_1$	☐$_2$	☐$_3$
30. Muscle tension	☐$_0$	☐$_1$	☐$_2$	☐$_3$
31. Jaw clenching/tight jaw	☐$_0$	☐$_1$	☐$_2$	☐$_3$
32. Burning, prickling or tingling sensation	☐$_0$	☐$_1$	☐$_2$	☐$_3$
33. Somatic energy, vibrations, or currents through your body	☐$_0$	☐$_1$	☐$_2$	☐$_3$
34. Muscle twitching	☐$_0$	☐$_1$	☐$_2$	☐$_3$
35. Involuntary eye movements (e.g., eye wiggles)	☐$_0$	☐$_1$	☐$_2$	☐$_3$
36. Blurred vision	☐$_0$	☐$_1$	☐$_2$	☐$_3$
37. Sensitivity to cold/feeling cold	☐$_0$	☐$_1$	☐$_2$	☐$_3$
38. Impaired gait/balance (e.g., difficulty walking, unsteadiness while standing or walking)	☐$_0$	☐$_1$	☐$_2$	☐$_3$
39. Dry mouth	☐$_0$	☐$_1$	☐$_2$	☐$_3$
40. Excessive thirst	☐$_0$	☐$_1$	☐$_2$	☐$_3$
41. Frequent urination or urge to urinate	☐$_0$	☐$_1$	☐$_2$	☐$_3$

M-SET Acute Treatment – X minutes Post-Treatment

Date of Assessment	_ _ / _ _ _ / _ _	Participant Initials	__ __ __	Participant ID	

Instructions: Rate items 42 to 47 based on the individual's self-report and observed behaviour since the medication was administered. Items 48 to 50 are rated on the basis of observed behaviour and speech.

**Symptoms**<br>Tick relevant items and provide further details in the **Clinical Notes** (e.g., pre-existing conditions, treatments, etc.)	**Not At All**	**Severity**<br>**Mild –** transient and easily tolerated<br>**Moderate –** caused discomfort and/or interference with usual activities<br>**Severe –** caused significant discomfort and/or considerable interference with usual activities		
		**Mild**	**Moderate**	**Severe**
42. Hostility (e.g., argumentative, aggressive, angry)	☐0	☐1	☐2	☐3
43. Grandiosity (e.g., feeling like you had special powers or abilities that others don't recognise, or that you might be somebody rich or famous?)	☐0	☐1	☐2	☐3
44. Suspiciousness (e.g., belief that other persons have acted maliciously or with bad intent)	☐0	☐1	☐2	☐3
45. Hallucinations (e.g., seeing, hearing, smelling or tasting things that are not present in reality)	☐0	☐1	☐2	☐3
46. Unusual thought content (e.g., delusions, ideas of reference/persecution)	☐0	☐1	☐2	☐3
47. Disorientation (e.g., does not comprehend situations or communications, confusion regarding person, place, or time)	☐0	☐1	☐2	☐3
48. Conceptual disorganisation (e.g., degree to which speech is confused, disconnected, vague or disorganised)	☐0	☐1	☐2	☐3
49. Tension (e.g., observable signs of physical tension, `nervousness' and agitation)	☐0	☐1	☐2	☐3
50. Motor hyperactivity (e.g., increase in energy level evidenced in more frequent movement and/or rapid speech)	☐0	☐1	☐2	☐3
51. Other (please specify):	☐0	☐1	☐2	☐3

Drug Effects Questionnaire

Instructions: "Please draw a mark on the line to show how strongly you are feeling each of the following effects right now."

1. How much did you feel any medication effect?

   NOT AT ALL |————————————| EXTREMELY

2. How much did you feel high or intoxicated?

   NOT AT ALL |————————————| EXTREMELY

3. How much did you dislike the medication effects?

   NOT AT ALL |————————————| EXTREMELY

4. How much did you like the medication effects?

   NOT AT ALL |————————————| EXTREMELY

5. How much did you want more of the medication you took?

   NOT AT ALL |————————————| EXTREMELY

M-SET Acute Treatment – X minutes Post-Treatment

Date of Assessment	_ _ / _ _ _ / _ _	Participant Initials	__ __ __	Participant ID	

Acute Treatment Physiological Measures			
Time	_ _ : _ _		
1. Blood pressure - systolic	________mmHg	☐-9	Missing
2. Blood pressure - diastolic	________mmHg	☐-9	Missing
3. Pulse	________BPM	☐-9	Missing
4. Temperature	________°C.	☐-9	Missing

Clinical Notes

Unless otherwise indicated, this form was completed by:
Signature: *Date:* D D / M M M / Y Y

M-SET Acute Treatment – Pre-Supp Dose

Date of Assessment	__ / ___ / __	Participant Initials	__ __ __	Participant ID	

Pre-Supplementary Dose: Symptoms Today

Rate items 1 to 40 based on the patient's self-report, using the following prompt: "Have you experienced any of the following symptoms today, since the medication was administered?

If yes, how severe were they?"

**Symptoms**<br>**Check relevant items and severity,** providing further details as necessary in the **Clinician Notes** (e.g., any other observations during treatment)	Not at all	Severity<br>**Mild** – transient and easily tolerated<br>**Moderate** – caused discomfort and/or interference with usual activities<br>**Severe** – caused significant discomfort and/or considerable interference with usual activities		
		**Mild**	**Moderate**	**Severe**
1. Anxiety	☐0	☐1	☐2	☐3
2. Fear of losing self-control	☐0	☐1	☐2	☐3
3. Panic attacks	☐0	☐1	☐2	☐3
4. Restlessness	☐0	☐1	☐2	☐3
5. Irritable mood	☐0	☐1	☐2	☐3
6. Low/depressed mood	☐0	☐1	☐2	☐3
7. Rumination (i.e., repeated negative thoughts that are hard to control)	☐0	☐1	☐2	☐3
8. Suicidal thoughts	☐0	☐1	☐2	☐3
9. Repeated, disturbing memories, thoughts, or images of a stressful experience from the past	☐0	☐1	☐2	☐3
10. Feeling threatened	☐0	☐1	☐2	☐3
11. Trouble concentrating	☐0	☐1	☐2	☐3
12. Thoughts and/or actions feeling slowed down or sped up	☐0	☐1	☐2	☐3
13. Experiencing your surroundings as strange and/or weird	☐0	☐1	☐2	☐3
14. Things seeming to be unreal or dreamlike	☐0	☐1	☐2	☐3
15. Your sense of your own body changed (e.g., felt unusually large or small)	☐0	☐1	☐2	
16. Objects looking different to what you would expect (e.g., distorted or unreal)	☐0	☐1	☐2	☐3
17. Things seem to be taking much longer or much less time than expected (e.g., as if time is passing slowly/standing still or there is a lifetime in a moment)	☐0	☐1	☐2	☐3
18. Sounds almost disappearing or becoming much stronger than you would have expected	☐0	☐1	☐2	☐3
19. Things seeming very real, as if there is a special sense of clarity	☐0	☐1	☐2	☐3
20. Feeling numb	☐0	☐1	☐2	☐3
21. Fear that you might or have said too much (over-disclosure, sharing of information you would rather have kept private)	☐0	☐1	☐2	☐3
22. Drowsiness	☐0	☐1	☐2	☐3
23. Fatigue	☐0	☐1	☐2	☐3
24. Feeling weak	☐0	☐1	☐2	☐3
25. Dizziness	☐0	☐1	☐2	☐3
26. Nausea and/or vomiting	☐0	☐1	☐2	☐3
27. Lower that typical appetite	☐0	☐1	☐2	☐3
28. Headache or migraine	☐0	☐1	☐2	☐3
29. Muscle tension	☐0	☐1	☐2	☐3
30. Jaw clenching/tight jaw	☐0	☐1	☐2	☐3
31. Burning, prickling or tingling sensations	☐0	☐1	☐2	☐3
32. Somatic energy, vibrations, or currents through your body	☐0	☐1	☐2	☐3
33. Muscle twitching	☐0	☐1	☐2	☐3
34. Involuntary eye movements (e.g., eye wiggles)	☐0	☐1	☐2	☐3
35. Blurred vision	☐0	☐1	☐2	☐3
36. Sensitivity to cold/feeling cold	☐0	☐1	☐2	☐3
37. Impaired gait/balance (e.g., difficulty walking, unsteadiness while standing or walking)	☐0	☐1	☐2	☐3
38. Dry mouth	☐0	☐1	☐2	☐3
39. Excessive thirst	☐0	☐1	☐2	☐3
40. Frequent urination or urge to urinate	☐0	☐1	☐2	☐3

M-SET Acute Treatment – Pre-Supp Dose

Date of Assessment	_ _ / _ _ _ / _ _	Participant Initials	__ __ __	Participant ID	

Instructions: Rate items 42 to 47 based on the individual's self-report and observed behaviour since the medication was administered. Items 48 to 50 are rated on the basis of observed behaviour and speech.

**Symptoms** Tick relevant items and provide further details in the **Clinical Notes** (e.g., pre-existing conditions, treatments, etc.)	**Not At All**	**Severity** **Mild –** transient and easily tolerated **Moderate –** caused discomfort and/or interference with usual activities **Severe –** caused significant discomfort and/or considerable interference with usual activities		
		**Mild**	**Moderate**	**Severe**
42. Hostility (e.g., argumentative, aggressive, angry)	☐0	☐1	☐2	☐3
43. Grandiosity (e.g., feeling like you had special powers or abilities that others don't recognise, or that you might be somebody rich or famous?)	☐0	☐1	☐2	☐3
44. Suspiciousness (e.g., belief that other persons have acted maliciously or with bad intent)	☐0	☐1	☐2	☐3
45. Hallucinations (e.g., seeing, hearing, smelling or tasting things that are not present in reality)	☐0	☐1	☐2	☐3
46. Unusual thought content (e.g., delusions, ideas of reference/persecution)	☐0	☐1	☐2	☐3
47. Disorientation (e.g., does not comprehend situations or communications, confusion regarding person, place, or time)	☐0	☐1	☐2	☐3
48. Conceptual disorganisation (e.g., degree to which speech is confused, disconnected, vague or disorganised)	☐0	☐1	☐2	☐3
49. Tension (e.g., observable signs of physical tension, `nervousness' and agitation)	☐0	☐1	☐2	☐3
50. Motor hyperactivity (e.g., increase in energy level evidenced in more frequent movement and/or rapid speech)	☐0	☐1	☐2	☐3
51. Other (please specify):	☐0	☐1	☐2	☐3

Drug Effects Questionnaire

Instructions: This questionnaire asks about how you are feeling after taking the substance that was given to you. Please draw a mark on the line to show how strongly you are feeling each of the following effects **right now**. You can mark anywhere on the line, but please draw a vertical line (one that goes straight up and down).

1. Do you **FEEL** a drug effect right now?

   NOT AT ALL |————————| EXTREMELY

2. Are you **HIGH** right now?

   NOT AT ALL |————————| EXTREMELY

3. Do you **DISLIKE** any of the effects you are feeling right now?

   NOT AT ALL |————————| EXTREMELY

4. Do you **LIKE** any of the effects you are feeling right now?

   NOT AT ALL |————————| EXTREMELY

5. Would you like **MORE** of the drug you took, right now?

   NOT AT ALL |————————| EXTREMELY

Pre-Supplementary Dose Physiological Measures

Time	_ _ : _ _		
1. **Blood pressure - systolic**	________mmHg	☐-9	Missing
2. **Blood pressure - diastolic**	________mmHg	☐-9	Missing
3. **Pulse**	________BPM	☐-9	Missing
4. **Temperature**	________°C.	☐-9	Missing

M-SET Acute Treatment – Pre-Supp Dose

Date of Assessment	_ _ / _ _ _ / _ _	Participant Initials	__ __ __	Participant ID	

Overall Tolerability<br>**Instructions:** Please complete when considering whether to administer a supplementary dose. Consider both subjective patient reports and objective observations, including symptoms and physiological measures.	
1. Unlikely to tolerate supplementary dose.	☐1
2. Likely to tolerate supplementary dose.	☐2

Treatment Administration Details	
**1. Time supplementary dosing**	_ _ : _ _ ☐-9 n/a
**2. Dose in mg**	________mg ☐-9 n/a
**3. If supplementary dose was not provided, please provide the reason**	☐-3 n/a<br>☐1 Could not tolerate last dose<br>☐2 Other. Please specify: ______________________________<br>______________________________
**Approved for dosing:**<br>***Signature:*** ***Date:*** D D / M M / Y Y	

Clinical Notes

**Unless otherwise indicated, this form was completed by:**<br>***Signature:*** ***Date:*** D D / M M M / Y Y

M-SET Acute Treatment – End of Treatment

Date of Assessment	__ / ___ / __	Participant Initials	__ __ __	Participant ID	

End of Treatment: Symptoms Today
Only rate for symptoms that have emerged **after** treatment today (i.e. new onset of symptoms or exacerbation of existing symptoms). If necessary, provide further details in the **Clinical Notes**.

Rate items 1 to 40 based on the patient's self-report, using the following prompt: "Are you still experiencing any of the following symptoms?"

Symptoms<br>**Tick the relevant items within each category.** Further details can be documented by the clinician in **Clinical Notes** (e.g., any other observations during measurement)	<u>RESOLVED</u> at end of treatment?		
	**No, not resolved**	**Yes, resolved**	**n/a**
1. Anxiety	☐$_0$	☐$_1$	☐$_{-3}$
2. Fear of losing self-control	☐$_0$	☐$_1$	☐$_{-3}$
3. Panic attacks	☐$_0$	☐$_1$	☐$_{-3}$
4. Restlessness	☐$_0$	☐$_1$	☐$_{-3}$
5. Irritable mood	☐$_0$	☐$_1$	☐$_{-3}$
6. Low/depressed mood	☐$_0$	☐$_1$	☐$_{-3}$
7. Rumination (i.e., repeated negative thoughts that are hard to control)	☐$_0$	☐$_1$	☐$_{-3}$
8. Suicidal thoughts	☐$_0$	☐$_1$	☐$_{-3}$
9. Repeated, disturbing memories, thoughts, or images of a stressful experience from the past	☐$_0$	☐$_1$	☐$_{-3}$
10. Feeling threatened	☐$_0$	☐$_1$	☐$_{-3}$
11. Trouble concentrating	☐$_0$	☐$_1$	☐$_{-3}$
12. Thoughts and/or actions feeling slowed down or sped up	☐$_0$	☐$_1$	☐$_{-3}$
13. Experiencing your surroundings as strange and/or weird	☐$_0$	☐$_1$	☐$_{-3}$
14. Things seeming to be unreal or dreamlike	☐$_0$	☐$_1$	☐$_{-3}$
15. Your sense of your own body changed (e.g., felt unusually large or small)	☐$_0$	☐$_1$	☐$_{-3}$
16. Objects looking different to what you would expect (e.g., distorted or unreal)	☐$_0$	☐$_1$	☐$_{-3}$
17. Things seem to be taking much longer or much less time than expected (e.g., as if time is passing slowly/standing still or there is a lifetime in a moment)	☐$_0$	☐$_1$	☐$_{-3}$
18. Sounds almost disappearing or becoming much stronger than you would have expected	☐$_0$	☐$_1$	☐$_{-3}$
19. Things seeming very real, as if there is a special sense of clarity	☐$_0$	☐$_1$	☐$_{-3}$
20. Feeling numb	☐$_0$	☐$_1$	☐$_{-3}$
21. Fear that you might or have said too much (over-disclosure, sharing of information you would rather have kept private)	☐$_0$	☐$_1$	☐$_{-3}$
22. Drowsiness	☐$_0$	☐$_1$	☐$_{-3}$
23. Fatigue	☐$_0$	☐$_1$	☐$_{-3}$
24. Feeling weak	☐$_0$	☐$_1$	☐$_{-3}$
25. Dizziness	☐$_0$	☐$_1$	☐$_{-3}$
26. Nausea and/or vomiting	☐$_0$	☐$_1$	☐$_{-3}$
27. Lower that typical appetite	☐$_0$	☐$_1$	☐$_{-3}$
28. Headache or migraine	☐$_0$	☐$_1$	☐$_{-3}$
29. Muscle tension	☐$_0$	☐$_1$	☐$_{-3}$
30. Jaw clenching/tight jaw	☐$_0$	☐$_1$	☐$_{-3}$
31. Burning, prickling or tingling sensations	☐$_0$	☐$_1$	☐$_{-3}$
32. Somatic energy, vibrations, or currents through your body	☐$_0$	☐$_1$	☐$_{-3}$
33. Muscle twitching	☐$_0$	☐$_1$	☐$_{-3}$
34. Involuntary eye movements (e.g., eye wiggles)	☐$_0$	☐$_1$	☐$_{-3}$
35. Blurred vision	☐$_0$	☐$_1$	☐$_{-3}$
36. Sensitivity to cold/feeling cold	☐$_0$	☐$_1$	☐$_{-3}$
37. Impaired gait/balance (e.g., difficulty walking, unsteadiness while standing or walking)	☐$_0$	☐$_1$	☐$_{-3}$
38. Dry mouth	☐$_0$	☐$_1$	☐$_{-3}$
39. Excessive thirst	☐$_0$	☐$_1$	☐$_{-3}$
40. Frequent urination or urge to urinate	☐$_0$	☐$_1$	☐$_{-3}$

M-SET Acute Treatment – End of Treatment

Date of Assessment	_ _ / _ _ _ / _ _	Participant Initials	__ __ __	Participant ID	

**Instructions:** Rate items 41 to 46 based on the patient's self-report and observed behaviour. Items 47 to 49 are rated on the basis of observed behaviour and speech.			
**Symptoms**<br>**Tick the relevant items within each category.** Further details can be documented by the clinician in **Clinical Notes** (e.g., any other observations during measurement)	**<u>RESOLVED</u> at X minutes?**		
	**No, not resolved**	**Yes, resolved**	**n/a**
41. Hostility (e.g., argumentative, aggressive, angry)	☐$_0$	☐$_1$	☐$_{-3}$
42. Grandiosity (e.g., feeling like you had special powers or abilities that others don't recognise, or that you might be somebody rich or famous?)	☐$_0$	☐$_1$	☐$_{-3}$
43. Suspiciousness (e.g., belief that other persons have acted maliciously or with bad intent)	☐$_0$	☐$_1$	☐$_{-3}$
44. Hallucinations (e.g., seeing, hearing, smelling or tasting things that are not present in reality)	☐$_0$	☐$_1$	☐$_{-3}$
45. Unusual thought content (e.g., delusions, ideas of reference/persecution)	☐$_0$	☐$_1$	☐$_{-3}$
46. Disorientation (e.g., does not comprehend situations or communications, confusion regarding person, place, or time)	☐$_0$	☐$_1$	☐$_{-3}$
47. Conceptual disorganisation (e.g., degree to which speech is confused, disconnected, vague or disorganised)	☐$_0$	☐$_1$	☐$_{-3}$
48. Tension (e.g., observable signs of physical tension, `nervousness' and/or agitation)	☐$_0$	☐$_1$	☐$_{-3}$
49. Motor hyperactivity (e.g., increase in energy level evidenced in more frequent movement and/or rapid speech)	☐$_0$	☐$_1$	☐$_{-3}$
50. Other (please specify):	☐$_0$	☐$_1$	☐$_{-3}$

Drug Effects Questionnaire

Instructions: This questionnaire asks about how you are feeling after taking the substance that was given to you. Please draw a mark on the line to show how strongly you are feeling each of the following effects **<u>right now</u>**. You can mark anywhere on the line, but please draw a vertical line (one that goes straight up and down).

1. Do you **FEEL** a drug effect right now?

   NOT AT ALL |————————————————| EXTREMELY

2. Are you **HIGH** right now?

   NOT AT ALL |————————————————| EXTREMELY

3. Do you **DISLIKE** any of the effects you are feeling right now?

   NOT AT ALL |————————————————| EXTREMELY

4. Do you **LIKE** any of the effects you are feeling right now?

   NOT AT ALL |————————————————| EXTREMELY

5. Would you like **MORE** of the drug you took, right now?

   NOT AT ALL |————————————————| EXTREMELY

M-SET Acute Treatment – End of Treatment

Date of Assessment	_ _ / _ _ _ / _ _	Participant Initials	__ __ __	Participant ID	

First Discharge Assessment Physiological Measures			
Time	_ _ : _ _		
1. **Blood pressure - systolic**	________mmHg	☐-9	Missing
2. **Blood pressure - diastolic**	________mmHg	☐-9	Missing
3. **Pulse**	________BPM	☐-9	Missing
4. **Temperature**	________°C.	☐-9	Missing

First Discharge Assessment Orientation
Instructions: Record answer if incorrect

Time	_ _ : _ _		
	Incorrect	Correct	Answer if incorrect
1. Name	☐0	☐1	
2. Date of birth	☐0	☐1	
3. Age	☐0	☐1	
4. Year	☐0	☐1	
5. Month	☐0	☐1	
6. Date	☐0	☐1	
7. Day	☐0	☐1	
8. Place	☐0	☐1	
9. Suburb	☐0	☐1	
Score	__ / 9		

First Discharge Assessment Discharge Checklist
Instructions: If discharge criteria not met, complete second discharge assessment at a later time (e.g., after 30 mins).

Discharge Checklist	First Discharge Assessment	
	No	Yes
1. Orientation score ≥ pre-treatment orientation score	☐0	☐1
2. Blood pressure < 120% Pre-treatment	☐0	☐1
3. Heart rate < 120% Pre-treatment	☐0	☐1
4. Can walk unassisted	☐0	☐1
5. Feeling physically well	☐0	☐1
6. Alert, calm, and comfortable (i.e., not distressed)	☐0	☐1
7. Participant meets all criteria for discharge	☐0	☐1

Overall Tolerability
Instructions: Please complete if the participant meets criteria for discharge on first assessment. If the participant requires a second discharge assessment, please complete the Overall Tolerability and Time of Discharge box on page 8. Select one answer prior to discharge. Consider both subjective patient reports and objective observations, including symptoms and physiological measures.

1. Unlikely to tolerate dose escalation.	☐1
2. Likely to tolerate dose escalation.	☐2

Time of Discharge

Time	_ _ : _ _

Clinical Notes

Unless otherwise indicated, this form was completed by:
Signature: ***Date:*** D D / M M M / Y Y

M-SET Acute Treatment – End of Treatment

Date of Assessment	__ / ___ / __	Participant Initials	__ __ __	Participant ID	

(If needed) Second Discharge Assessment Physiological Measures			
Time	__ : __		
5. **Blood pressure - systolic**	________mmHg	☐-9	Missing
6. **Blood pressure - diastolic**	________mmHg	☐-9	Missing
7. **Pulse**	________BPM	☐-9	Missing
8. **Temperature**	________°C.	☐-9	Missing

(If needed) Second Discharge Assessment Orientation<br>**Instructions:** Record answer if incorrect			
Time	__ : __		
	Incorrect	Correct	Answer if incorrect
10. Name	☐0	☐1	
11. Date of birth	☐0	☐1	
12. Age	☐0	☐1	
13. Year	☐0	☐1	
14. Month	☐0	☐1	
15. Date	☐0	☐1	
16. Day	☐0	☐1	
17. Place	☐0	☐1	
18. Suburb	☐0	☐1	
Score	__ / 9		

(If needed) Second Discharge Assessment Discharge Checklist		
**Discharge Checklist**	**First Discharge Assessment**	
	**No**	**Yes**
8. Orientation score ≥ pre-treatment orientation score	☐0	☐1
9. Blood pressure < 120% Pre-treatment	☐0	☐1
10. Heart rate < 120% Pre-treatment	☐0	☐1
11. Can walk unassisted	☐0	☐1
12. Feeling physically well	☐0	☐1
13. Alert, calm, and comfortable (i.e., not distressed)	☐0	☐1
14. Participant meets all criteria for discharge	☐0	☐1

(If needed) Overall Tolerability

Instructions: Only complete here if the participant is discharged after the second discharge assessment. If the participant is discharged after first discharge assessment, please complete Overall Tolerability and Time of Discharge box on page 6.
Select one answer prior to discharge. Consider both subjective patient reports and objective observations, including symptoms and physiological measures.

1. Unlikely to tolerate dose escalation.	☐1
2. Likely to tolerate dose escalation.	☐2

**Time of Discharge**	
Time	__ : __

M-SET – Follow-Up

Date of Assessment	__ / ___ / __	Participant Initials	__ __ __	Participant ID	

Instructions: The MSET Follow-up Questionnaire should be completed by a clinician or researcher at follow-up, after receiving MDMA-assisted psychotherapy.

Rate items 1 to 58 based on the patient's self-report, using the following prompt: "Have you experienced the following symptoms since your last medication-assisted psychotherapy session? If yes, how severe were they?"

Symptoms<br>Check relevant items, providing further details as necessary in the Clinical Notes	Not Since Last Medication Session	Severity<br>Mild – transient and easily tolerated<br>Moderate – caused discomfort and/or interference with usual activities<br>Severe – caused significant discomfort and/or considerable interference with usual activities		
		Mild	Moderate	Severe
1. Anxiety	☐$_0$	☐$_1$	☐$_2$	☐$_3$
2. Fear of losing self-control	☐$_0$	☐$_1$	☐$_2$	☐$_3$
3. Panic attacks	☐$_0$	☐$_1$	☐$_2$	☐$_3$
4. Restlessness	☐$_0$	☐$_1$	☐$_2$	☐$_3$
5. Irritable mood	☐$_0$	☐$_1$	☐$_2$	☐$_3$
6. Low/depressed mood	☐$_0$	☐$_1$	☐$_2$	☐$_3$
7. Anguish or despair	☐$_0$	☐$_1$	☐$_2$	☐$_3$
8. Unprompted inconsolable crying	☐$_0$	☐$_1$	☐$_2$	☐$_3$
9. Rumination (i.e., repeated negative thoughts that are hard to control)	☐$_0$	☐$_1$	☐$_2$	☐$_3$
10. Suicidal thoughts	☐$_0$	☐$_1$	☐$_2$	☐$_3$
11. Suicidal behaviour (e.g., suicide attempt, interrupted or aborted attempt, preparatory acts)	☐$_0$	☐$_1$	☐$_2$	☐$_3$
12. Thoughts of self-harm and/or intentional self-harm, without suicidal intent	☐$_0$	☐$_1$	☐$_2$	☐$_3$
13. Repeated, disturbing memories, thoughts, or images of a stressful experience from the past	☐$_0$	☐$_1$	☐$_2$	☐$_3$
14. Feeling threatened	☐$_0$	☐$_1$	☐$_2$	☐$_3$
15. Feeling disconnected from your friends, family, or social group	☐$_0$	☐$_1$	☐$_2$	☐$_3$
16. Feeling that it's hard to connect with others or that socializing is a real effort	☐$_0$	☐$_1$	☐$_2$	☐$_3$
17. Lack of drive or motivation to pursue goals previously valued as meaningful	☐$_0$	☐$_1$	☐$_2$	☐$_3$
18. Impaired ability to do normal work/study, potentially putting employment/education at risk	☐$_0$	☐$_1$	☐$_2$	☐$_3$
19. Difficulties with memory and/or concentration	☐$_0$	☐$_1$	☐$_2$	☐$_3$
20. Difficulty making even the smallest decision	☐$_0$	☐$_1$	☐$_2$	☐$_3$
21. Thoughts and/or actions feeling slowed down or sped up	☐$_0$	☐$_1$	☐$_2$	☐$_3$
22. Experienced your surroundings as strange and/or weird	☐$_0$	☐$_1$	☐$_2$	☐$_3$
23. Things seeming to be unreal or dreamlike	☐$_0$	☐$_1$	☐$_2$	☐$_3$
24. Feeling separated from what is happening around you (e.g., as if you are in the movie or a play)	☐$_0$	☐$_1$	☐$_2$	☐$_3$
25. Feeling disconnected from your own body or looking at things from outside your body	☐$_0$	☐$_1$	☐$_2$	☐$_3$
26. Your sense of your own body changed (e.g., felt unusually large or small)	☐$_0$	☐$_1$	☐$_2$	☐$_3$
27. Objects looked different to what you would expect (e.g., distorted or unreal)	☐$_0$	☐$_1$	☐$_2$	☐$_3$
28. Seeing things as if you were in a tunnel, or looking through a wide-angle photographic lens	☐$_0$	☐$_1$	☐$_2$	☐$_3$
29. Things seemed to take much longer than you would have expected (e.g., as if time is passing slowly/standing still)	☐$_0$	☐$_1$	☐$_2$	☐$_3$
30. Things seemed to be happening very quickly, as if there is a lifetime in a moment	☐$_0$	☐$_1$	☐$_2$	☐$_3$
31. Sounds almost disappeared or became much stronger than you would have expected	☐$_0$	☐$_1$	☐$_2$	☐$_3$
32. Things seeming very real, as if there is a special sense of clarity	☐$_0$	☐$_1$	☐$_2$	☐$_3$
33. Unprompted hysterical laughter	☐$_0$	☐$_1$	☐$_2$	☐$_3$
34. Drowsiness	☐$_0$	☐$_1$	☐$_2$	☐$_3$
35. Fatigue	☐$_0$	☐$_1$	☐$_2$	☐$_3$
36. Feeling weak	☐$_0$	☐$_1$	☐$_2$	☐$_3$
37. Dizziness	☐$_0$	☐$_1$	☐$_2$	☐$_3$
38. Insomnia (e.g., difficulty falling asleep, staying asleep and/or other sleep problems)	☐$_0$	☐$_1$	☐$_2$	☐$_3$
39. Nightmares	☐$_0$	☐$_1$	☐$_2$	☐$_3$
40. Feeling the need for much less or much more sleep than usual	☐$_0$	☐$_1$	☐$_2$	☐$_3$
41. Diarrhoea	☐$_0$	☐$_1$	☐$_2$	☐$_3$
42. Nausea and/or vomiting	☐$_0$	☐$_1$	☐$_2$	☐$_3$
43. Lower than typical appetite	☐$_0$	☐$_1$	☐$_2$	☐$_3$
44. Lack of interest in sex	☐$_0$	☐$_1$	☐$_2$	☐$_3$
45. Physical pain	☐$_0$	☐$_1$	☐$_2$	☐$_3$

M-SET – Follow-Up

Date of Assessment	_ _ / _ _ _ / _ _	Participant Initials	__ __ __	Participant ID	

If yes, where?: ____________				
46. Headache or migraine	☐0	☐1	☐2	☐3
47. Muscle tension	☐0	☐1	☐2	☐3
48. Jaw clenching/tight jaw	☐0	☐1	☐2	☐3
49. Burning, prickling or tingling sensation	☐0	☐1	☐2	☐3
50. Somatic energy, vibrations, or currents through your body	☐0	☐1	☐2	☐3
51. Muscle twitching	☐0	☐1	☐2	☐3
52. Involuntary eye movements (e.g., eye wiggles)	☐0	☐1	☐2	☐3
53. Blurred vision	☐0	☐1	☐2	☐3
54. Sensitivity to cold/feeling cold	☐0	☐1	☐2	☐3
55. Impaired gait/balance (e.g., difficulty walking, unsteadiness while standing or walking)	☐0	☐1	☐2	☐3
56. Dry mouth	☐0	☐1	☐2	☐3
57. Excessive thirst	☐0	☐1	☐2	☐3
58. Frequent urination or urge to urinate	☐0	☐1	☐2	☐3

Instructions: Rate items 59 to 64 based on the patient's self-report since their last medication-assisted psychotherapy session and observed behaviour. Items 65 to 68 are rated on the basis of observed behaviour and speech.

Symptoms<br>Check relevant items, providing further details as necessary in the **Clinical Notes** (e.g., pre-existing conditions, treatments, etc.)	Not Since Last Medication Session	Severity<br>Mild – transient and easily tolerated<br>Moderate – caused discomfort and/or interference with usual activities<br>Severe – caused significant discomfort and/or considerable interference with usual activities		
		Mild	Moderate	Severe
59. Unusually elevated mood (e.g., exaggerated feeling of well-being, cheerfulness, euphoria and optimism)	☐0	☐1	☐2	☐3
60. Grandiosity (e.g., feeling like you had special powers or abilities that others don't recognise, or that you might be somebody rich or famous?)	☐0	☐1	☐2	☐3
61. Suspiciousness (e.g., belief that other persons have acted maliciously or with bad intent)	☐0	☐1	☐2	☐3
62. Hallucinations (e.g., seeing, hearing, smelling or tasting things that are not present in reality)	☐0	☐1	☐2	☐3
63. Unusual thought content (e.g., delusions, ideas of reference/persecution)	☐0	☐1	☐2	☐3
64. Disorientation (e.g., does not comprehend situations or communications, confusion regarding person, place, or time)	☐0	☐1	☐2	☐3
65. Conceptual disorganisation (e.g., degree to which speech is confused, disconnected, vague or disorganised)	☐0	☐1	☐2	☐3
66. Tension (e.g., observable signs of physical tension, `nervousness' and/or agitation)	☐0	☐1	☐2	☐3
67. Motor hyperactivity (e.g., increase in energy level evidenced in more frequent movement and/or rapid speech)	☐0	☐1	☐2	☐3
68. Abnormal mannerisms and/or posturing (e.g., grimacing, rocking, nodding, postures which are clearly uncomfortable or inappropriate)	☐0	☐1	☐2	☐3
69. Other (please specify):	☐0	☐1	☐2	☐3

M-SET – Follow-Up

Date of Assessment	_ _ / _ _ _ / _ _	Participant Initials	__ __ __	Participant ID	

Physical Examination			
1. Blood pressure - systolic	________mmHg	☐-9	Missing
2. Blood pressure - diastolic	________mmHg	☐-9	Missing
3. Pulse	________BPM	☐-9	Missing
4. Respiration Rate	________(breaths per minute)	☐-9	Missing
5. Temperature	________°C.	☐-9	Missing

Clinical Notes

Unless otherwise indicated, this form was completed by:
Signature: *Date:* D D / M M M / Y Y
