# Supplementary material for: Development of the MDMA-Assisted Psychotherapy Side Effects Tool (M-SET): a Delphi study
Source: BMJ Open. 2026 May 11;16(5):e105630. doi: 10.1136/bmjopen-2025-105630 (PMC13182389; doi:10.1136/bmjopen-2025-105630)
Supplement: online supplemental file 2 [file bmjopen-16-5-s002.pdf]

Table S1: CREDES (Conducting and REporting DElphi Studies) guidance

**Rationale for the choice of the Delphi technique**	**Location**
1. *Justification.* The choice of the Delphi technique as a method of systematically collating expert consultation and building consensus needs to be well justified. When selecting the method to answer a particular research question, it is important to keep in mind its constructivist nature	p 3
**Planning and design**	
2. *Planning and process.* The Delphi technique is a flexible method and can be adjusted to the respective research aims and purposes. Any modifications should be justified by a rationale and be applied systematically and rigorously	p 4
3. *Definition of consensus.* Unless not reasonable due to the explorative nature of the study, an a priori criterion for consensus should be defined. This includes a clear and transparent guide for action on (a) how to proceed with certain items or topics in the next survey round, (b) the required threshold to terminate the Delphi process and (c) procedures to be followed when consensus is (not) reached after one or more iterations	p 4-5
**Study conduct**	
4. *Informational input.* All material provided to the expert panel at the outset of the project and throughout the Delphi process should be carefully reviewed and piloted in advance in order to examine the effect on experts' judgements and to prevent bias	n/a
5. *Prevention of bias.* Researchers need to take measures to avoid directly or indirectly influencing the experts' judgements. If one or more members of the research team have a conflict of interest, entrusting an independent researcher with the main coordination of the Delphi study is advisable	n/a
6. *Interpretation and processing of results.* Consensus does not necessarily imply the 'correct' answer or judgement; (non)consensus and stable disagreement provide informative insights and highlight differences in perspectives concerning the topic in question	p 10
7. *External validation.* It is recommended to have the final draft of the resulting guidance on best practice in palliative care reviewed and approved by an external board or authority before publication and dissemination	n/a
**Reporting**	
8. *Purpose and rationale.* The purpose of the study should be clearly defined and demonstrate the appropriateness of the use of the Delphi technique as a method to achieve the research aim. A rationale for the choice of the Delphi technique as the most suitable method needs to be provided	p 1-2
9. *Expert panel.* Criteria for the selection of experts and transparent information on recruitment of the expert panel, socio-demographic details including information on expertise regarding the topic in question, (non)response and response rates over the ongoing iterations should be reported	p 4-5
10. *Description of the methods.* The methods employed need to be comprehensible; this includes information on preparatory steps (How was available evidence on the topic in question synthesised?), piloting of material and survey instruments, design of the survey instrument(s), the number and design of survey rounds, methods of data analysis, processing and synthesis of experts' responses to inform the subsequent survey round and methodological decisions taken by the research team throughout the process	p 3-5
11. *Procedure.* Flow chart to illustrate the stages of the Delphi process, including a preparatory phase, the actual 'Delphi rounds', interim steps of data processing and analysis, and concluding steps	Figures 1 & 2
12. *Definition and attainment of consensus.* It needs to be comprehensible to the reader how consensus was achieved throughout the process, including strategies to deal with non-consensus	p 4-5
13. *Results.* Reporting of results for each round separately is highly advisable in order to make the evolving of consensus over the rounds transparent. This includes figures showing the average group response, changes between rounds, as well as any modifications of the survey instrument such as deletion, addition or modification of survey items based on previous rounds	p 6-7
14. *Discussion of limitations.* Reporting should include a critical reflection of potential limitations and their impact of the resulting guidance	p 10
15. *Adequacy of conclusions.* The conclusions should adequately reflect the outcomes of the Delphi study with a view to the scope and applicability of the resulting practice guidance	p 11
16. *Publication and dissemination.* The resulting guidance on good practice in palliative care should be clearly identifiable from the publication, including recommendations for transfer into practice and implementation. If the publication does not allow for a detailed presentation of either the resulting practice guidance or the methodological features of the applied Delphi technique, or both, reference to a more detailed presentation elsewhere should be made (e.g. availability of the full guideline from the authors or online; publication of a separate paper reporting on methodological details and particularities of the process (e.g. persistent disagreement and controversy on certain issues)). A dissemination plan should include endorsement of the guidance by professional associations and health care authorities to facilitate implementation	p 10-11

Table S2. Items included in the Round One item pool and their source

Table S2.1. Screening Questionnaire

Item	Source
**Possible contraindications for MDMA-assisted psychotherapy**	Systematic Review
Active or past psychotic disorder, borderline personality disorder, dissociative identity disorder or eating disorder	
Family history of psychotic disorder	
High blood pressure (hypertension)	
Heart/cardiovascular condition	
Seizures/stroke/head injury/neurological disorder	
Glaucoma	
Liver condition	
Kidney condition	
Diabetes	
Hepatitis	
Hyponatremia or hyperthermia	
Problematic alcohol or drug use	
Epilepsy	
Immunological diseases or recent infection (within 4 weeks)	
For females: Current pregnancy	
For females: Current breastfeeding	
Allergies.<br>If yes, please list: ____	
**History of MDMA Use**	Systematic Review and KSET

Have you ever received or used MDMA for any reason? Please tick all that apply	
When did you last take MDMA, Ecstasy or Molly?	
How many times in total (lifetime)?	
Route (e.g., oral, intranasal/'snorting')	
Highest dose received	
Did you have any negative or unpleasant reactions?	
Have you ever craved MDMA? If "yes", please specify how recently?______________________	
**Current use of medications or supplements**	Systematic Review
Current use of medications or supplements? If "yes" please list all medications and supplements below	
**Physical Examination**	Systematic Review and KSET
Weight	
Height	
Blood pressure – systolic	
Blood pressure – diastolic	
Pulse	
Respiration Rate	
Oxygen Saturation	
**Clinical Investigations**	Systematic Review and KSET
Liver Function Tests	
Full Blood Examination	
Thyroid Function Test (TFT)	
Electrocardiogram (ECG)	

Table S2.2 Baseline and Follow-Up Questionnaires

Item	Source
**Symptoms**	
Anxiety	Systematic Review
Drowsiness, fatigue and/or weakness	
Low/depressed mood	
Lack of appetite	
Insomnia	
Irritable mood	
Headache	
Difficulties with memory and/or concentration	
Nausea and/or vomiting	
Jaw clenching/tight jaw	
Rumination	
Dry mouth	
Muscle tension	
Restlessness	
Perspiration	
Impaired gait/balance	
Sensitivity to cold/feeling cold	
Dizziness	
Diarrhea	

Thirst	
Panic attack	
Suicidal thoughts	
Tic	
Lack of libido	
Burning or prickling sensation	
Anguish or despair	
Nightmares	
Somatic pains	
Involuntary eye movements	
Blurred vision	
Frequent urination or urge to urinate	
Stress	
Muscle twitching	
Self-harm	
Heavy legs	
Hallucinations (e.g., seeing, hearing, smelling or tasting things that are not present in reality)	BPRS
Unusual thought content (e.g., delusions, ideas of reference/persecution)	
Suspiciousness (e.g., belief that other persons have acted maliciously or with bad intent)	
Grandiosity (e.g., exaggerated self-opinion, belief that one has special abilities or powers or identity as someone rich or famous)	
Elevated mood (e.g., exaggerated feeling of well-being, cheerfulness, euphoria and optimism)	
Motor hyperactivity (e.g., increase in energy level evidenced in more frequent movement and/or rapid speech)	

Conceptual disorganisation (e.g., degree to which speech is confused, disconnected, vague or disorganised)	
Tension (e.g., observable signs of physical tension, `nervousness' and agitation)	
Disorientation (e.g., does not comprehend situations or communications, confusion regarding person, place, or time)	
Abnormal mannerisms and/or posturing (e.g., grimacing, rocking, nodding, postures which are clearly uncomfortable or inappropriate)	
Hostility (e.g., argumentative, aggressive, angry)	
Bizarre behaviour (e.g., inappropriate giggling/laughter, talking to self, fixated staring)	
Self-neglect (e.g., Hygiene, appearance, or eating behaviour below usual expectations, below socially acceptable standards or life threatening)	
Things moving in slow motion	CADSS
Things seeming to be unreal or dreamlike	
Feeling separated from what is happening around you (e.g., as if you are in the movie or a play)	
Feeling as if you are looking at things from outside of your body	
Feeling as if you are watching situations as an observer or a spectator	
Feeling disconnected from your own body	
Your sense of your own body changed (e.g., felt unusually large or small)	
Objects looked different to what you would expect (e.g., distorted, unreal)	
Colours seemed to be diminished in intensity	
Seeing things as if you were in a tunnel, or looking through a wide-angle photographic lens	
Things seemed to take much longer than you would have expected (e.g., as if time is passing slowly/standing still)	
Things seemed to be happening very quickly, as if there is a lifetime in a moment	
Sounds almost disappeared or became much stronger than you would have expected	
Things seemed very real, as if there is a special sense of clarity	
Colours seemed much brighter than you would have expected	

Repeated, disturbing memories, thoughts, or images of a stressful experience from the past	IUE
Feeling disconnected from your friends, family, or social group	
Feeling like it's hard to connect to others or that socializing is a real effort	
Feeling the need for much less or much more sleep than usual	
Unprompted inconsolable crying	
Unprompted hysterical laughter	
Lack of drive or motivation to pursue goals previously valued as meaningful	
An impaired ability to do one's normal work/study, potentially putting one's employment/education at risk	
Somatic energy, vibrations, or currents through one's body	
Fear of losing self-control	OAV
Experienced your surroundings as strange and weird	
Afraid without being able to say exactly why	
Felt threatened	
Felt isolated from everything and everyone	
Body felt numb, dead and weird	
Difficulty making even the smallest decision	
Thoughts and actions were slowed down	5D-ASC
Felt numb	

BPRS: Brief Psychiatric Rating Scale; CADSS: Clinician-Administered Dissociative States Scale; IUE: Inventory of Unusual Experiences among meditators; OAV: Altered States of Consciousness Rating Scale; 5D-ASC: 5-Dimensional Altered States of Consciousness Rating Scale

Table S2.3 Follow-Up Questionnaire only

**Item**	**Source**
**Physical Examination**	Systematic Review and KSET

Blood pressure – systolic	
Blood pressure - diastolic	
Pulse	
Respiration Rate	
Oxygen Saturation	
**Clinical Investigations**	Systematic Review and KSET
Liver Function Tests	
Full Blood Examination	
Thyroid Function Test (TFT)	
Electrocardiogram (ECG)	

Table S2.3 Acute Questionnaire

**Item**	**Source**
**Acute Treatment Physiological Measures**	Systematic Review
Blood pressure – systolic	
Blood pressure – diastolic	
Pulse	
Temperature	
**Drug Effects Questionnaire**	DEQ
How much did you feel any medication effect?	
How much did you feel high or intoxicated?	
How much did you dislike the medication effects?	
How much did you like the medication effects?	
How much did you want more of the medication you took?	
**Symptoms**	
Anxiety	Systematic Review
Panic	
Fatigue and/or weakness	
Headache	

Jaw clenching/tight jaw	
Lack of appetite	
Dizziness	
Perspiration	
Restlessness	
Muscle tension	
Sensitivity to cold/feeling cold	
Thirst	
Difficulties with memory and/or concentration	
Low/depressed mood	
Impaired gait/balance	
Irritable mood	
Dry mouth	
Nausea and/or vomiting	
Suicidal thoughts	
Drowsiness	
Need more sleep	
Rumination/increased private worries	
Anguish or despair	
Insomnia	
Somatic pains	
Dilated pupils	
Involuntary eye movements	
Blurred vision	
Frequent urination or urge to urinate	
Stress	
Muscle twitching	
Tic	
Heavy legs	
Burning or prickling sensation	

Hallucinations (e.g., seeing, hearing, smelling or tasting things that are not present in reality)	BPRS
Unusual thought content (e.g., delusions, ideas of reference/persecution)	
Suspiciousness (e.g., belief that other persons have acted maliciously or with bad intent)	
Grandiosity (e.g., exaggerated self-opinion, belief that one has special abilities or powers or identity as someone rich or famous)	
Elevated mood (e.g., exaggerated feeling of well-being, cheerfulness, euphoria and optimism)	
Motor hyperactivity (e.g., increase in energy level evidenced in more frequent movement and/or rapid speech)	
Conceptual disorganisation (e.g., degree to which speech is confused, disconnected, vague or disorganised)	
Tension (e.g., observable signs of physical tension, `nervousness' and agitation)	
Disorientation (e.g., does not comprehend situations or communications, confusion regarding person, place, or time)	
Abnormal mannerisms and/or posturing (e.g., grimacing, rocking, nodding, postures which are clearly uncomfortable or inappropriate)	
Hostility (e.g., argumentative, aggressive, angry)	
Bizarre behaviour (e.g., inappropriate giggling/laughter, talking to self, fixated staring)	
Things moving in slow motion	CADSS
Things seeming unreal or dreamlike	
Feeling separated from what is happening around you (e.g., as if you are in the movie or a play)	
Feeling as if you are looking at things from outside of your body	
Feeling as if you are watching the situation as an observer or a spectator	
Feeling disconnected from your own body	
Your sense of your own body changed (e.g., felt unusually large or small)	
Objects looking different to what you would expect (e.g., distorted, unreal)	
Colours seeming to be diminished in intensity	
Seeing things as if you were in a tunnel, or looking through a wide-angle photographic lens	
Things seeming to take much longer than you would have expected (e.g., as if time is passing slowly/standing still)	
Things seeming to be happen very quickly, as if there is a lifetime in a moment	

Sounds almost disappearing or becoming much stronger than you would have expected	
Things seeming very real, as if there is a special sense of clarity	
Colours seeming much brighter than you would have expected	
Repeated, disturbing memories, thoughts, or images of a stressful experience from the past	IUE
Unprompted inconsolable crying	
Unprompted hysterical laughter	
Somatic energy, vibrations, or currents through your body	
Fear of losing self-control	OAV
Experiencing your surroundings as strange and weird	
Afraid without being able to say exactly why	
Feeling threatened	
Feeling isolated from everything and everyone	
Body feeling numb, dead and weird	
Difficulty making even the smallest decision	
Thoughts and actions being slowed down	5D-ASC
Feeling numb	

BPRS: Brief Psychiatric Rating Scale; CADSS: Clinician-Administered Dissociative States Scale; DEQ: Drug Effects Questionnaire; IUE: Inventory of Unusual Experiences among meditators; OAV: Altered States of Consciousness Rating Scale; 5D-ASC: 5-Dimensional Altered States of Consciousness Rating Scale

Table S3. Delphi Round One Results Summary

	Keep	Discard	Modify	OUTCOME	R2 Item	Keep	Discard	Modify	Add
**SCREENING QUESTIONNAIRE**									
**Possible contraindications for MDMA-psychotherapy**									
Active or past psychotic disorder, borderline personality disorder, dissociative identity disorder or eating disorder	45%	0%	55%	Active or past psychotic disorder (e.g., schizophrenia, bipolar-1 with mania), borderline personality disorder and/or panic attacks	X			X	
Family history of psychotic disorder	36%	9%	55%	First-degree relative with an active or past psychotic disorder	X			X	
High blood pressure (hypertension)	36%	0%	64%	Uncontrolled hypertension	X			X	
Heart/cardiovascular condition	73%	0%	27%	Heart/cardiovascular condition. If yes, please specify:	X			X	
Seizures/stroke/head injury/neurological disorder	64%	0%	36%	Seizures, including epilepsy. If yes, controlled or uncontrolled?<br>Cerebrovascular condition, including previous stroke. If yes, please specify:<br>Head injury. If yes, please specify:	X<br>X<br>X			X	 X<br>X
Glaucoma	73%	0%	27%	Glaucoma. If yes, controlled or uncontrolled?	X			X	
Liver condition	64%	9%	27%	Liver condition. If yes, please specify:	X			X	
Kidney condition	64%	9%	27%	Kidney condition. If yes, please specify:	X			X	
Diabetes	73%	18%	9%	Diabetes	X				
Hepatitis	73%	9%	18%	Included under liver condition item			X		
Hyponatremia or hyperthermia	64%	9%	27%	Previous experience of Hyponatremia	X			X	

	Keep	Discard	Modify	OUTCOME	R2 Item	Keep	Discard	Modify	Add
Problematic alcohol or drug use	36%	9%	55%	Alcohol or drug use during the past 6 months. If yes, please specify: Frequency ____ Quantity ____	X			X	
Epilepsy	64%	0%	36%	Included under seizures item			X		
Immunological diseases or recent infection (within 4 weeks)	55%	9%	36%	Autoimmune condition. If yes, please specify:<br>Infection with significant impact on physical health within 4 weeks. If yes, please specify:	X<br>X			X	 X
For females: Current pregnancy	82%	0%	18%	Current pregnancy or potential to become pregnant		X		X	
For females: Current breastfeeding	91%	0%	9%	Current breastfeeding		X		X	
Allergies If yes, please list:	64%	9%	27%	Allergies (food and/or drug)<br>If yes, please list:		X[1]		X	
**History of MDMA Use**									
Have you ever received or used MDMA for any reason? Please tick all that apply<br>No previous use (please continue on to Allergies section)<br>Psychiatric treatment. If yes, was it helpful?<br>Recreational use (e.g., Ecstasy, Molly)<br>Other. Please specify ________________________________	82%	0%	18%	Have you ever received or used MDMA? (Check all that apply)<br>No previous use<br>Psychiatric treatment, with professional support. If yes, was it helpful?<br>Therapeutic use in non-medical/non-research setting. If yes, was it helpful?<br>Recreational use (e.g., Ecstasy, Molly)<br>Other. Please specify ________________________________		X		X	
When did you last take MDMA, Ecstasy or Molly?	100%	0%	0%	When did you last take MDMA, Ecstasy or Molly?		X			
How many times in total (lifetime)?	82%	0%	18%	How many occasions in total (lifetime)?<br>How many occasions in the last year?		X<br>X			

	Keep	Discard	Modify	OUTCOME	R2 Item	Keep	Discard	Modify	Add
Route (e.g., oral, intranasal/'snorting')	64%	0%	36%	How did you take it (e.g., by mouth, intranasal/'snorting', IV, suppository)<br>In what form was MDMA used? (e.g., crystals, pills)	X<br><br>X			X	 <br>X
Highest dose received	55%	9%	36%	Highest dose you think you've received/taken at one time<br><100mg<br>100-200mg<br>>200mg<br>Don't know<br><br>How sure were you that what you took was actually MDMA? (/10)	X<br><br><br><br><br><br>X			X	 <br><br><br><br><br>X
Did you have any negative or unpleasant reactions?	82%	0%	18%	Did you have any negative or unpleasant reactions?		X			
Have you ever craved MDMA? Yes / No<br>If “yes”, please specify how recently?______________________	64%	9%	27%	Have you ever craved or had an intense desire or urge to take MDMA that made it difficult for you to think of anything else? If yes, please specify how recently:	X			X	
**Current use of medications or supplements**									
Current use of medications or supplements?	73%	0%	27%	Current/past 6 months use of medications or supplements?<br>Dose: __________<br>Frequency of use: __________<br>Years on medication/supplement: __________	X			X	
**Physical Examination**									
Weight	100%	0%	0%	Weight		X			
Height	100%	0%	0%	Height		X			
Blood pressure – systolic	100%	0%	0%	Blood pressure - systolic		X			
Blood pressure – diastolic	100%	0%	0%	Blood pressure - diastolic		X			
Pulse	100%	0%	0%	Pulse		X			

	Keep	Discard	Modify	OUTCOME	R2 Item	Keep	Discard	Modify	Add
Respiration Rate	82%	18%	0%	Respiration Rate		X			
Oxygen Saturation	64%	36%	0%	Oxygen Saturation	X				
**Clinical Investigations**									
Liver Function Tests	82%	9%	9%	Liver Function Tests (e.g., ALT, AST, ALP, GGT)		X		X	
Full Blood Examination	64%	9%	27%	Full Blood Examination (at clinical discretion)	X			X	
Thyroid Function Test (TFT)	64%	0%	36%	Thyroid Function Test (TFT) (at clinical discretion)	X			X	
Electrocardiogram (ECG)	82%	9%	9%	Electrocardiogram (ECG/EKG)		X		X	
**Additional items**									
Malignant hyperthermia				Additional item	X				X
Have you taken MDMA and experienced no effects from it?				Additional item	X				X
History of Hallucinogen Persisting Perception Disorder				Additional item	X				X
History of visual distortions (e.g., visual snow)				Additional item	X				X
Lubben Social Network Scale–6 (LSNS-6)<br>FAMILY: Considering the people to whom you are related by birth, marriage, adoption, etc… How many relatives do you see or hear from at least once a month?				Additional section	X				X

	Keep	Discard	Modify	OUTCOME	R2 Item	Keep	Discard	Modify	Add
How many relatives do you feel at ease with that you can talk about private matters How many relatives do you feel close to such that you could call on them for help?<br>FRIENDSHIPS: Considering all of your friends including those who live in your neighbourhood<br>How many of your friends do you see or hear from at least once a month?<br>How many friends do you feel at ease with that you can talk about private matters?<br>How many friends do you feel close to such that you could call on them for help?									
Number of years of formal education				Additional item	X				X
**BASELINE AND FOLLOW-UP QUESTIONNAIRE**									
**Symptoms**									
Anxiety	91%	0%	9%	Anxiety		X			
Drowsiness, fatigue and/or weakness	82%	0%	18%	Drowsiness<br>Fatigue<br>Feeling weak		X<br>X<br>X		 <br>X	
Low/depressed mood	100%	0%	0%	Low/depressed mood		X			
Lack of appetite	91%	0%	9%	Lower than typical appetite		X		X	
Insomnia	82%	0%	18%	Insomnia (e.g., difficulty falling asleep, staying asleep and/or other sleep problems)		X		X	
Irritable mood	91%	9%	0%	Irritable mood		X			
Headache	82%	0%	18%	Headache or migraine		X		X	

	Keep	Discard	Modify	OUTCOME	R2 Item	Keep	Discard	Modify	Add
Difficulties with memory and/or concentration	100%	0%	0%	Difficulties with memory and/or concentration		X			
Nausea and/or vomiting	100%	0%	0%	Nausea and/or vomiting		X			
Jaw clenching/tight jaw	100%	0%	0%	Jaw clenching/tight jaw		X			
Rumination	73%	0%	27%	Rumination (e.g., repeated negative thoughts or overthinking)	X			X	
Dry mouth	100%	0%	0%	Dry mouth		X			
Muscle tension	100%	0%	0%	Muscle tension		X			
Restlessness	91%	9%	0%	Restlessness		X			
Perspiration	70%	10%	20%	Excessive perspiration	X			X	
Impaired gait/balance	91%	0%	9%	Impaired gait/balance (e.g., difficulty walking, unsteadiness while standing or walking)		X		X	
Sensitivity to cold/feeling cold	90%	10%	0%	Sensitivity to cold/feeling cold		X			
Dizziness	100%	0%	0%	Dizziness		X			
Diarrhea	82%	9%	9%	Diarrhoea		X		X	
Thirst	91%	0%	9%	Excessive thirst		X		X	

	Keep	Discard	Modify	OUTCOME	R2 Item	Keep	Discard	Modify	Add
Panic attack	82%	0%	18%	Panic attacks		X		X	
Suicidal thoughts	100%	0%	0%	Suicidal thoughts		X			
Tic	55%	18%	27%	Tics (uncontrolled sudden, repetitive movement or sound that can be hard to control)	X			X	
Lack of libido	82%	0%	18%	Lack of interest in sex		X		X	
Burning or prickling sensation	82%	9%	9%	Burning, prickling or tingling sensations		X		X	
Anguish or despair	82%	18%	0%	Anguish or despair		X			
Nightmares	100%	0%	0%	Nightmares		X			
Somatic pains	55%	9%	36%	Physical pain. If yes, where?	X			X	
Involuntary eye movements	82%	18%	0%	Involuntary eye movements (e.g., eye wiggles)		X		X	
Blurred vision	82%	18%	0%	Blurred vision		X			
Frequent urination or urge to urinate	82%	18%	0%	Frequent urination or urge to urinate		X			
Stress	64%	27%	9%	Stress (e.g., feeling tense, unable to relax, touchy)	X			X	
Muscle twitching	91%	9%	0%	Muscle twitching		X			

	Keep	Discard	Modify	OUTCOME	R2 Item	Keep	Discard	Modify	Add
Self-harm	64%	0%	36%	Thoughts of self-harm and/or intentional self-harm, without suicidal intent	X			X	
Heavy legs	73%	27%	0%	Heavy legs	X				
Hallucinations (e.g., seeing, hearing, smelling or tasting things that are not present in reality)	82%	9%	9%	Hallucinations (e.g., seeing, hearing, smelling or tasting things that are not present in reality)		X			
Unusual thought content (e.g., delusions, ideas of reference/persecution)	91%	0%	9%	Unusual thought content (e.g., delusions, ideas of reference/persecution)		X			
Suspiciousness (e.g., belief that other persons have acted maliciously or with bad intent)	91%	9%	0%	Suspiciousness (e.g., belief that other persons have acted maliciously or with bad intent)		X			
Grandiosity (e.g., exaggerated self-opinion, belief that one has special abilities or powers or identity as someone rich or famous)	73%	9%	18%	Grandiosity (e.g., feeling like there is anything special about you, like you had special powers or abilities or that you might be somebody rich or famous?)<br>Clinical notes/instructions: Clinician-rated, based on individual's self-report Endorsing this item as Moderate to Severe in Baseline Questionnaire should be considered cause for further investigation/potential exclusion	X			X	
Elevated mood (e.g., exaggerated feeling of well-being, cheerfulness, euphoria and optimism)	82%	0%	18%	Unusually elevated mood (e.g., exaggerated feeling of well-being, cheerfulness, euphoria and optimism)		X		X	
Motor hyperactivity (e.g., increase in energy level evidenced in more frequent movement and/or rapid speech)	91%	9%	0%	Motor hyperactivity (e.g., increase in energy level evidenced in more frequent movement and/or rapid speech)		X			
Conceptual disorganisation (e.g., degree to which speech is confused, disconnected, vague or disorganised)	82%	18%	0%	Conceptual disorganisation (e.g., degree to which speech is confused, disconnected, vague or disorganised)		X			
Tension (e.g., observable signs of physical tension, `nervousness' and agitation)	82%	18%	0%	Tension (e.g., observable signs of physical tension, `nervousness' and agitation)		X			
Disorientation (e.g., does not comprehend situations or communications, confusion regarding person, place, or time)	91%	0%	9%	Disorientation (e.g., does not comprehend situations or communications, confusion regarding person, place, or time)		X			
Abnormal mannerisms and/or posturing (e.g., grimacing, rocking, nodding, postures which are clearly uncomfortable or inappropriate)	73%	18%	9%	Abnormal mannerisms and/or posturing (e.g., grimacing, rocking, nodding, postures which are clearly uncomfortable or inappropriate)	X			X	

	Keep	Discard	Modify	OUTCOME	R2 Item	Keep	Discard	Modify	Add
				Clinical notes/instructions: Clinician-rated, based on observed behaviour					
Hostility (e.g., argumentative, aggressive, angry)	73%	9%	18%	Hostility (e.g., argumentative, aggressive, angry)	X				
Bizarre behaviour (e.g., inappropriate giggling/laughter, talking to self, fixated staring)	64%	18%	18%	Bizarre behaviour (e.g., inappropriate giggling/laughter, talking to self, fixated staring)<br>Clinical notes/instructions: Clinician-rated, based on observed behaviour	X			X	
Self-neglect (e.g., Hygiene, appearance, or eating behaviour below usual expectations, below socially acceptable standards or life threatening)	73%	18%	9%	Self-neglect (e.g., Hygiene, appearance, or eating behaviour below usual expectations, below socially acceptable standards or life threatening)<br>Clinical notes/instructions: Clinician-rated, based on observed behaviour	X			X	
Things moving in slow motion	64%	27%	9%	Things moving in slow motion or sped up	X			X	
Things seeming to be unreal or dreamlike	91%	9%	0%	Things seeming to be unreal or dreamlike		X			
Feeling separated from what is happening around you (e.g., as if you are in the movie or a play)	80%	20%	0%	Feeling separated from what is happening around you (e.g., as if you are in the movie or a play)		X			
Feeling as if you are looking at things from outside of your body	82%	18%	0%	Feeling disconnected from your own body or looking at things from outside your body	X			X	
Feeling as if you are watching situations as an observer or a spectator	73%	18%	9%	Combined with other dissociation items			X		
Feeling disconnected from your own body	82%	9%	9%	Combined with above item			X		
Your sense of your own body changed (e.g., felt unusually large or small)	91%	9%	0%	Your sense of your own body changed (e.g., felt unusually large or small)		X			
Objects looked different to what you would expect (e.g., distorted, unreal)	91%	9%	0%	Objects looked different to what you would expect (e.g., distorted, unreal)		X			

	Keep	Discard	Modify	OUTCOME	R2 Item	Keep	Discard	Modify	Add
Colours seemed to be diminished in intensity	64%	18%	18%	Colours seemed to be diminished in intensity or much brighter than you would have expected	X			X	
Colours seemed much brighter than you would have expected	64%	27%	9%	Combined with above item			X		
Seeing things as if you were in a tunnel, or looking through a wide-angle photographic lens	82%	18%	0%	Seeing things as if you were in a tunnel, or looking through a wide-angle photographic lens		X			
Things seemed to take much longer than you would have expected (e.g., as if time is passing slowly/standing still)	91%	9%	0%	Things seemed to take much longer than you would have expected (e.g., as if time is passing slowly/standing still)		X			
Things seemed to be happening very quickly, as if there is a lifetime in a moment	91%	9%	0%	Things seemed to be happening very quickly, as if there is a lifetime in a moment		X			
Sounds almost disappeared or became much stronger than you would have expected	82%	18%	0%	Sounds almost disappeared or became much stronger than you would have expected		X			
Things seemed very real, as if there is a special sense of clarity	91%	0%	9%	Things seeming very real, as if there is a special sense of clarity		X		X	
Repeated, disturbing memories, thoughts, or images of a stressful experience from the past	91%	0%	9%	Repeated, disturbing memories, thoughts, or images of a stressful experience from the past		X			
Feeling disconnected from your friends, family, or social group	82%	9%	9%	Feeling disconnected from friends, family, or social group		X		X	
Feeling like it’s hard to connect to others or that socializing is a real effort	82%	9%	9%	Feeling that it’s hard to connect with others or that socializing is a real effort		X		X	
Feeling the need for much less or much more sleep than usual	91%	9%	0%	Feeling the need for much less or much more sleep than usual		X			
Unprompted inconsolable crying	82%	18%	0%	Unprompted inconsolable crying		X			
Unprompted hysterical laughter	82%	18%	0%	Unprompted hysterical laughter		X			

	Keep	Discard	Modify	OUTCOME	R2 Item	Keep	Discard	Modify	Add
Lack of drive or motivation to pursue goals previously valued as meaningful	82%	18%	0%	Lack of drive or motivation to pursue goals previously valued as meaningful		X			
An impaired ability to do one's normal work/study, potentially putting one's employment/education at risk	82%	18%	0%	Impaired ability to do normal work/study, potentially putting employment/education at risk		X		X	
Somatic energy, vibrations, or currents through one's body	82%	18%	0%	Somatic energy, vibrations, or currents through your body		X		X	
Fear of losing self-control	100%	0%	0%	Fear of losing self-control		X			
Experienced your surroundings as strange and weird	82%	9%	9%	Experienced your surroundings as strange and/or weird		X		X	
Afraid without being able to say exactly why	73%	27%	0%	Afraid without being able to say exactly why	X				
Felt threatened	91%	0%	9%	Feeling threatened		X		X	
Felt isolated from everything and everyone	64%	27%	9%	Felt isolated from everything and everyone	X				
Body felt numb, dead and weird	73%	9%	18%	Body felt numb, dead or weird	X			X	
Difficulty making even the smallest decision	82%	18%	0%	Difficulty making even the smallest decisions		X		X	
Thoughts and actions were slowed down	70%	10%	20%	Thoughts and/or actions were slowed down or sped up	X			X	
Felt numb	73%	9%	18%	Felt emotionally detached or numb	X			X	
**Physical Examination (Follow-up only)**									

	Keep	Discard	Modify	OUTCOME	R2 Item	Keep	Discard	Modify	Add
Blood pressure - systolic	100%	0%	0%	Blood pressure - systolic		X			
Blood pressure - diastolic	100%	0%	0%	Blood pressure - diastolic		X			
Pulse	100%	0%	0%	Pulse		X			
Respiration Rate	82%	18%	0%	Respiration Rate		X			
Oxygen Saturation	64%	36%	0%	Oxygen Saturation	X				
**Clinical Investigations (Follow-up only)**									
Liver Function Tests	55%	9%	36%	Liver Function Tests (at clinical discretion; e.g., ALT, AST, ALP, GGT)	X			X	
Full Blood Examination	36%	27%	36%	Full Blood Examination (at clinical discretion)	X			X	
Thyroid Function Test (TFT)	36%	27%	36%	Thyroid Function Test (TFT; at clinical discretion)	X			X	
Electrocardiogram (ECG)	36%	18%	45%	Electrocardiogram (ECG; at clinical discretion)	X			X	
**Additional items**									
Inability or difficulty to urinate				Additional item	X				X
Cold or other infection				Additional item	X				X
Temperature (at clinical discretion)				Additional item	X				X

	Keep	Discard	Modify	OUTCOME	R2 Item	Keep	Discard	Modify	Add
**ACUTE QUESTIONNAIRE**									
**Symptoms**									
Anxiety	90%	0%	10%	Anxiety		X			
Panic	90%	0%	10%	Panic attacks		X		X	
Fatigue and/or weakness	80%	10%	10%	Fatigue<br>Feeling weak		X<br>X		 X	
Headache	90%	0%	10%	Headache or migraine		X		X	
Jaw clenching/tight jaw	100%	0%	0%	Jaw clenching/tight jaw		X			
Lack of appetite	100%	0%	0%	Lower that typical appetite		X		X	
Dizziness	100%	0%	0%	Dizziness		X			
Perspiration	70%	10%	20%	Excessive perspiration	X			X	
Restlessness	90%	10%	0%	Restlessness		X			
Muscle tension	90%	10%	0%	Muscle tension		X			
Sensitivity to cold/feeling cold	80%	10%	10%	Sensitivity to cold/feeling cold		X			
Thirst	80%	10%	10%	Excessive thirst		X		X	

	Keep	Discard	Modify	OUTCOME	R2 Item	Keep	Discard	Modify	Add
Difficulties with memory and/or concentrating	70%	10%	20%	Trouble concentrating<br>Difficulties with memory	X<br>X			X	 X
Low/depressed mood	100%	0%	0%	Low/depressed mood		X			
Impaired gait/balance	90%	0%	10%	Impaired gait/balance (e.g., difficulty walking, unsteadiness while standing or walking)		X		X	
Irritable mood	80%	20%	0%	Irritable mood		X			
Dry mouth	90%	10%	0%	Dry mouth		X			
Nausea and/or vomiting	90%	0%	10%	Nausea and/or vomiting		X			
Suicidal thoughts	100%	0%	0%	Suicidal thoughts		X			
Drowsiness	90%	10%	0%	Drowsiness		X			
Need more sleep	60%	30%	10%	Feeling tired	X			X	
Rumination/increased private worries	80%	10%	10%	Rumination (i.e., repeated negative thoughts that are hard to control)		X		X	
Anguish or despair	70%	30%	0%	Anguish or despair	X				
Insomnia	70%	0%	30%	Insomnia<br>Clinical notes/instructions: Administer only after drug effects	X			X	
Somatic pains	70%	10%	20%	Physical pain. If yes, where?	X			X	

	Keep	Discard	Modify	OUTCOME	R2 Item	Keep	Discard	Modify	Add
Dilated pupils	70%	10%	20%	Dilated pupils<br>Clinical notes/instructions: Clinician-rated, based on observation	X			X	
Involuntary eye movements	80%	10%	10%	Involuntary eye movements (e.g., eye wiggles)		X		X	
Blurred vision	90%	10%	0%	Blurred vision		X			
Frequent urination or urge to urinate	90%	10%	0%	Frequent urination or urge to urinate		X			
Stress	70%	30%	0%	Stress (e.g., feeling tense, unable to relax, touchy)	X			X	
Muscle twitching	90%	10%	0%	Muscle twitching		X			
Tic	60%	20%	20%	Tics (uncontrolled sudden, repetitive movement or sound that can be hard to control)	X			X	
Heavy legs	70%	30%	0%	Heavy legs	X				
Burning or prickling sensation	80%	10%	10%	Burning, prickling or tingling sensations		X		X	
Hallucinations (e.g., seeing, hearing, smelling or tasting things that are not present in reality)	90%	0%	10%	Hallucinations (e.g., seeing, hearing, smelling or tasting things that are not present in reality)		X			
Unusual thought content (e.g., delusions, ideas of reference/persecution)	80%	10%	10%	Unusual thought content (e.g., delusions, ideas of reference/persecution)		X			
Suspiciousness (e.g., belief that other persons have acted maliciously or with bad intent)	90%	10%	0%	Suspiciousness (e.g., belief that other persons have acted maliciously or with bad intent)		X			
Grandiosity (e.g., exaggerated self-opinion, belief that one has special abilities or powers or identity as someone rich or famous)	80%	10%	10%	Grandiosity (e.g., feeling like you had special powers or abilities that others don't recognise, or that you might be somebody rich or famous?)		X		X	

	Keep	Discard	Modify	OUTCOME	R2 Item	Keep	Discard	Modify	Add
Motor hyperactivity (e.g., increase in energy level evidenced in more frequent movement and/or rapid speech)	90%	10%	0%	Motor hyperactivity (e.g., increase in energy level evidenced in more frequent movement and/or rapid speech)		X			
Conceptual disorganisation (e.g., degree to which speech is confused, disconnected, vague or disorganised)	90%	10%	0%	Conceptual disorganisation (e.g., degree to which speech is confused, disconnected, vague or disorganised)		X			
Disorientation (e.g., does not comprehend situations or communications, confusion regarding person, place, or time)	80%	0%	20%	Disorientation (e.g., does not comprehend situations or communications, confusion regarding person, place, or time)		X			
Hostility (e.g., argumentative, aggressive, angry)	80%	20%	0%	Hostility (e.g., argumentative, aggressive, angry)		X			
Elevated mood (e.g., exaggerated feeling of well-being, cheerfulness, euphoria and optimism)	70%	20%	10%	Unusually elevated mood (e.g., exaggerated feeling of well-being, cheerfulness, euphoria and optimism)	X			X	
Tension (e.g., observable signs of physical tension, `nervousness' and agitation)	70%	20%	10%	Tension (e.g., observable signs of physical tension, nervousness and agitation)<br>Clinical notes/instructions: Clinician-rated, based on observed behaviour	X			X	
Abnormal mannerisms and/or posturing (e.g., grimacing, rocking, nodding, postures which are clearly uncomfortable or inappropriate)	70%	10%	20%	Abnormal mannerisms and/or posturing (e.g., grimacing, rocking, nodding, postures which are clearly uncomfortable or inappropriate)<br>Clinical notes/instructions: Clinician-rated, based on observed behaviour	X			X	
Bizarre behaviour (e.g., inappropriate giggling/laughter, talking to self, fixated staring)	60%	30%	10%	Bizarre behaviour (e.g., inappropriate giggling/laughter, talking to self, fixated staring)<br>Clinical notes/instructions: Clinician-rated, based on observed behaviour	X			X	
Things moving in slow motion	50%	30%	20%	Things moving in slow motion or sped up	X			X	
Feeling separated from what is happening around you (e.g., as if you are in the movie or a play)	70%	30%	0%	Feeling separated from what is happening around you (e.g., as if you are in the movie or a play)	X				
Feeling as if you are looking at things from outside of your body	60%	30%	10%	Feeling disconnected from your own body or looking at things from outside your body	X			X	
Feeling as if you are watching the situation as an observer or a spectator	60%	30%	10%	Combined with above item			X		

	Keep	Discard	Modify	OUTCOME	R2 Item	Keep	Discard	Modify	Add
Feeling disconnected from your own body	70%	20%	10%	Combined with above item			X		
Colours seeming to be diminished in intensity	60%	20%	20%	Colours seemed to be diminished in intensity or much brighter than you would have expected	X			X	
Colours seeming much brighter than you would have expected	60%	30%	10%	Combined with above item			X		
Seeing things as if you were in a tunnel, or looking through a wide-angle photographic lens	70%	30%	0%	Seeing things as if you were in a tunnel, or looking through a wide-angle photographic lens	X				
Things seeming to take much longer than you would have expected (e.g., as if time is passing slowly/standing still)	70%	20%	10%	Things seem to be taking much longer or much less time than expected (e.g., as if time is passing slowly/standing still or there is a lifetime in a moment)	X			X	
Things seeming to be happen very quickly, as if there is a lifetime in a moment	70%	20%	10%	Combined with above item			X		
Things seeming unreal or dreamlike	80%	20%	0%	Things seeming unreal or dreamlike		X			
Your sense of your own body changed (e.g., felt unusually large or small)	80%	20%	0%	Your sense of your own body changed (e.g., felt unusually large or small)		X			
Objects looking different to what you would expect (e.g., distorted, unreal)	80%	20%	0%	Objects looking different to what you would expect (e.g., distorted, unreal)		X			
Sounds almost disappearing or becoming much stronger than you would have expected	80%	20%	0%	Sounds almost disappearing or becoming much stronger than you would have expected		X			
Things seeming very real, as if there is a special sense of clarity	80%	20%	0%	Things seeming very real, as if there is a special sense of clarity		X			
Repeated, disturbing memories, thoughts, or images of a stressful experience from the past	80%	20%	0%	Repeated, disturbing memories, thoughts, or images of a stressful experience from the past		X			
Somatic energy, vibrations, or currents through your body	80%	20%	0%	Somatic energy, vibrations, or currents through your body		X			

	Keep	Discard	Modify	OUTCOME	R2 Item	Keep	Discard	Modify	Add
Fear of losing self-control	90%	10%	0%	Fear of losing self-control		X			
Experiencing your surroundings as strange and weird	80%	20%	0%	Experiencing your surroundings as strange and weird		X			
Feeling threatened	90%	0%	10%	Feeling threatened		X			
Feeling numb	80%	20%	0%	Feeling numb		X			
Unprompted inconsolable crying	70%	30%	0%	Unprompted inconsolable crying	X				
Unprompted hysterical laughter	70%	30%	0%	Unprompted hysterical laughter	X				
Afraid without being able to say exactly why	70%	20%	10%	Afraid without being able to say exactly why	X				
Feeling isolated from everything and everyone	60%	40%	0%	Feeling isolated from everything and everyone	X				
Body feeling numb, dead and weird	70%	20%	10%	Body feeling numb, dead or weird	X			X	
Difficulty making even the smallest decision	70%	30%	0%	Difficulty making even the smallest decision	X				
Thoughts and actions being slowed down	60%	20%	20%	Thoughts and/or actions being slowed down or sped up	X			X	
**Acute Treatment Physiological Measures**									
Blood pressure - systolic	100%	0%	0%	Blood pressure - systolic		X			

	Keep	Discard	Modify	OUTCOME	R2 Item	Keep	Discard	Modify	Add
Blood pressure - diastolic	100%	0%	0%	Blood pressure - diastolic		X			
Pulse	100%	0%	0%	Pulse		X			
Temperature	100%	0%	0%	Temperature		X			
**Drug Effects Questionnaire**									
How much did you feel any medication effect?	90%	0%	10%	Do you FEEL a drug effect right now?		X		X	
How much did you feel high or intoxicated?	90%	10%	0%	Are you HIGH right now?		X		X	
How much did you dislike the medication effects?	100%	0%	0%	Do you DISLIKE any of the effects you are feeling right now?		X		X	
How much did you like the medication effects?	100%	0%	0%	Do you LIKE any of the effects you are feeling right now?		X		X	
How much did you want more of the medication you took?	90%	10%	0%	Would you like MORE of the drug you took, right now?		X		X	
**Additional Items**									
Fear that you might or have said too much (over-disclosure, sharing of information you would rather have kept private)				Additional item	X				X

[1]Retained in error

Table S4. Delphi Round Two Results Summary

	Keep	Discard	Modify	Keep + Modify	OUTCOME	Keep	Discard	Modify	Add
**SCREENING QUESTIONNAIRE**									
**Relevant History and Comorbidities**									
Seizures, including epilepsy. If yes, controlled or uncontrolled?	100%	0%	0%	100%	Seizures, including epilepsy. If yes, controlled or uncontrolled?	X			
Cerebrovascular condition, including previous stroke. If yes, please specify:	100%	0%	0%	100%	Cerebrovascular condition, including previous stroke. If yes, please specify:	X			
Liver condition. If yes, please specify:	89%	0%	11%	100%	Liver condition. If yes, please specify:	X			
Active or past psychotic disorder (e.g., schizophrenia, bipolar-1 with mania), borderline personality disorder and/or panic attacks	56%	0%	44%	100%	Separate items:<br>Active or past psychotic disorder (e.g., schizophrenia)<br>Bipolar disorder<br>Borderline personality disorder<br>Panic attacks	 X		 X	 <br>X<br>X<br>X
First-degree relative with an active or past psychotic disorder	67%	22%	11%	78%	Discarded		X		
Uncontrolled hypertension	56%	0%	44%	100%	Uncontrolled hypertension	X			
Heart/cardiovascular condition. If yes, please specify:	78%	0%	22%	100%	Heart/cardiovascular condition. If yes, please specify:	X			
Head injury. If yes, please specify:	63%	25%	13%	75%	Discarded		X		
Glaucoma. If yes, controlled or uncontrolled?	78%	0%	22%	100%	Glaucoma. If yes, controlled or uncontrolled?	X			
Kidney condition. If yes, please specify:	78%	11%	11%	89%	Kidney condition. If yes, please specify:	X			
Diabetes	67%	22%	11%	78%	Discarded		X		

	Keep	Discard	Modify	Keep + Modify	OUTCOME	Keep	Discard	Modify	Add
Previous experience of Hyponatremia	38%	13%	50%	88%	Discarded		X		
Alcohol or drug use during the past 6 months.<br>If yes, please specify: Frequency ____ Quantity ____	44%	11%	44%	89%	Alcohol or drug use during the past 6 months<br>If yes, please specify:<br>Frequency: __________<br>Quantity: __________<br><br>Note: Further assessment for current Substance Use Disorder, including physiological dependence and capacity to comply with the treatment protocol may be required.	X		X	
Autoimmune condition. If yes, please specify:	78%	11%	11%	89%	Autoimmune condition (e.g., fibromyalgia, lupus, multiple sclerosis)<br>If yes, please specify:	X		X	
Infection with significant impact on physical health within 4 weeks. If yes, please specify:	78%	11%	11%	89%	Infection with significant impact on physical health within 4 weeks. If yes, please specify:	X			
Malignant hyperthermia	44%	22%	33%	78%	Discarded		X		
History of Hallucinogen Persisting Perception Disorder	56%	22%	22%	78%	Discarded		X		
History of visual distortions (e.g., visual snow)	56%	22%	22%	78%	Discarded		X		
Number of years of formal education	56%	33%	11%	67%	Discarded		X		
**History of MDMA Use**									
How did you take it? (e.g., by mouth, intranasal/'snorting', IV, suppository)	78%	0%	22%	100%	How did you take it (e.g., by mouth, intranasal/'snorting', IV, suppository) and in what form? (e.g., crystals, pills)	X		X	
In what form was MDMA used? (e.g., crystals, pills)	67%	11%	22%	89%	Combined with above item		X		

	Keep	Discard	Modify	Keep + Modify	OUTCOME	Keep	Discard	Modify	Add
Highest dose you think you've received/taken at one time<br><100mg<br>100-200mg<br>>200mg<br>Don't know	100%	0%	0%	100%	Highest dose you think you've received/taken at one time<br><100mg<br>100-200mg<br>>200mg<br>Don't know	X			
How sure were you that what you took was actually MDMA? (/10)	78%	11%	11%	89%	How sure were you that what you took was actually MDMA? (1 to 10; 1 = not sure at all; 10 = very sure)	X		X	
Have you ever craved or had an intense desire or urge to take MDMA that made it difficult for you to think of anything else? If yes, please specify how recently:	78%	11%	11%	89%	Have you ever craved or had an intense desire or urge to take MDMA?<br>If “yes”, how recently?	X		X	
Have you taken MDMA and experienced no effects from it?	44%	33%	22%	67%	Discarded		X		
**Current/past 6 months use of medications or supplements**									
Current or past 6 months use of medication or supplements<br>Dose ____<br>Frequency of use ____<br>Years on medication/supplement ____	89%	0%	11%	100%	Current or past 6 months use of medication or supplements<br>Dose ____<br>Frequency of use ____<br>Years on medication/supplement ____	X			
**Physical Examination**									
Oxygen Saturation	22%	78%	0%	22%	Discarded		X		
**Clinical Investigations (at clinical discretion)**									
Full Blood Examination (at clinical discretion)	63%	25%	13%	75%	Discarded		X		
Thyroid Function Test (TFT) (at clinical discretion)	78%	22%	0%	78%	Discarded		X		

	Keep	Discard	Modify	Keep + Modify	OUTCOME	Keep	Discard	Modify	Add
**Social Support**									
Lubben Social Network Scale–6 (LSNS-6) FAMILY: Considering the people to whom you are related by birth, marriage, adoption, etc… How many relatives do you see or hear from at least once a month? How many relatives do you feel at ease with that you can talk about private matters How many relatives do you feel close to such that you could call on them for help? FRIENDSHIPS: Considering all of your friends including those who live in your neighbourhood How many of your friends do you see or hear from at least once a month? How many friends do you feel at ease with that you can talk about private matters? How many friends do you feel close to such that you could call on them for help?	67%	22%	11%	78%	Discarded		X		
**BASELINE AND FOLLOW-UP QUESTIONNAIRE**									
**Symptoms**									
Thoughts of self-harm and/or intentional self-harm, without suicidal intent	89%	11%	0%	100%	Thoughts of self-harm and/or intentional self-harm, without suicidal intent	X			
Thoughts and/or actions were slowed down or sped up	100%	0%	0%	100%	Thoughts and/or actions were slowed down or sped up	X			
Heavy legs	11%	89%	0%	11%	Discarded		X		
Rumination (e.g., repeated negative thoughts or overthinking)	78%	0%	22%	100%	Rumination (i.e., repeated negative thoughts that are hard to control)	X		X	
Excessive perspiration	44%	56%	0%	44%	Discarded		X		
Tics (uncontrolled sudden, repetitive movement or sound that can be hard to control)	56%	44%	0%	56%	Discarded		X		
Physical pain. If yes, where?	63%	13%	25%	88%	Physical pain. If yes, where?	X			

	Keep	Discard	Modify	Keep + Modify	OUTCOME	Keep	Discard	Modify	Add
Stress (e.g., feeling tense, unable to relax, touchy)	44%	22%	33%	78%	Discarded		X		
Things moving in slow motion or sped up	33%	56%	11%	44%	Discarded		X		
Feeling disconnected from your own body or looking at things from outside your body	78%	11%	11%	89%	Feeling disconnected from your own body or looking at things from outside your body	X			
Colours seemed to be diminished in intensity or much brighter than you would have expected	67%	11%	22%	89%	Discarded		X		
Afraid without being able to say exactly why	56%	33%	11%	67%	Discarded		X		
Felt isolated from everything and everyone	78%	22%	0%	78%	Discarded		X		
Body felt numb, dead or weird	44%	44%	11%	56%	Discarded		X		
Felt emotionally detached or numb	67%	22%	11%	78%	Discarded		X		
Inability or difficulty to urinate	67%	33%	0%	67%	Discarded		X		
Cold or other infection	44%	44%	11%	56%	Discarded		X		
Abnormal mannerisms and/or posturing (e.g., grimacing, rocking, nodding, postures which are clearly uncomfortable or inappropriate)<br>Clinical notes/instructions: Clinician-rated, based on observed behaviour	88%	13%	0%	100%	Abnormal mannerisms and/or posturing (e.g., grimacing, rocking, nodding, postures which are clearly uncomfortable or inappropriate)<br>Clinical notes/instructions: Clinician-rated, based on observed behaviour	X			
Grandiosity (e.g., feeling like there is anything special about you, like you had special powers or abilities or that you might be somebody rich or famous?)<br>Clinical notes/instructions: Clinician-rated, based on individual's self-report. Endorsing this item as Moderate to Severe in Baseline	67%	11%	22%	89%	Grandiosity (e.g., feeling like there is anything special about you, like you had special powers or abilities or that you might be somebody rich or famous?)<br>Clinical notes/instructions: Clinician-rated, based on individual's self-report. Endorsing this item as Moderate to	X			

	Keep	Discard	Modify	Keep + Modify	OUTCOME	Keep	Discard	Modify	Add
Questionnaire should be considered cause for further investigation/potential exclusion					Severe in Baseline Questionnaire should be considered cause for further investigation/potential exclusion				
Hostility (e.g., argumentative, aggressive, angry)	33%	56%	11%	44%	Discarded		X		
Bizarre behaviour (e.g., inappropriate giggling/laughter, talking to self, fixated staring)<br>Clinical notes/instructions: Clinician-rated, based on observed behaviour	67%	33%	0%	67%	Discarded		X		
Self-neglect (e.g., Hygiene, appearance, or eating behaviour below usual expectations, below socially acceptable standards or life threatening)<br>Clinical notes/instructions: Clinician-rated, based on observed behaviour	67%	22%	11%	78%	Discarded		X		
**Physical Examination (Follow-up only)**									
Temperature (at clinical discretion)	88%	13%	0%	100%	Temperature	X			
Oxygen Saturation	22%	78%	0%	22%	Discarded		X		
**Clinical Investigations (Follow-up only)**									
Liver Function Tests (at clinical discretion; e.g., ALT, AST, ALP, GGT)	67%	22%	11%	78%	Discarded		X		
Full Blood Examination (at clinical discretion)	56%	33%	11%	67%	Discarded		X		
Thyroid Function Test (TFT; at clinical discretion)	75%	25%	0%	75%	Discarded		X		
Electrocardiogram (ECG; at clinical discretion)	78%	11%	11%	89%	Discarded		X		
**ACUTE QUESTIONNAIRE**									

	Keep	Discard	Modify	Keep + Modify	OUTCOME	Keep	Discard	Modify	Add
**Symptoms**									
Trouble concentrating	100%	0%	0%	100%	Trouble concentrating	X			
Tension (e.g., observable signs of physical tension, nervousness and agitation)<br>Clinical notes/instructions: Clinician-rated, based on observed behaviour	100%	0%	0%	100%	Tension (e.g., observable signs of physical tension, nervousness and agitation)<br>Clinical notes/instructions: Clinician-rated, based on observed behaviour	X			
Fear that you might or have said too much (over-disclosure, sharing of information you would rather have kept private)	89%	0%	11%	100%	Fear that you might or have said too much (over-disclosure, sharing of information you would rather have kept private)	X			
Excessive perspiration	78%	22%	0%	78%	Discarded		X		
Difficulties with memory	63%	25%	13%	75%	Discarded		X		
Feeling tired	44%	44%	11%	56%	Discarded		X		
Anguish or despair	44%	56%	0%	44%	Discarded		X		
Insomnia<br>Clinical notes/instructions: Administer only after drug effects	56%	22%	22%	78%	Discarded		X		
Physical pain. If yes, where?	78%	22%	0%	78%	Discarded		X		
Dilated pupils<br>Clinical notes/instructions: Clinician-rated, based on observation	56%	44%	0%	56%	Discarded		X		
Stress (e.g., feeling tense, unable to relax, touchy)	67%	0%	33%	100%	Discarded		X		
Tics (uncontrolled sudden, repetitive movement or sound that can be hard to control)	78%	22%	0%	78%	Discarded		X		

	Keep	Discard	Modify	Keep + Modify	OUTCOME	Keep	Discard	Modify	Add
Heavy legs	33%	67%	0%	33%	Discarded		X		
Unusually elevated mood (e.g., exaggerated feeling of well-being, cheerfulness, euphoria and optimism)	78%	22%	0%	78%	Discarded		X		
Abnormal mannerisms and/or posturing (e.g., grimacing, rocking, nodding, postures which are clearly uncomfortable or inappropriate)<br>Clinical notes/instructions: Clinician-rated, based on observed behaviour	78%	22%	0%	78%	Discarded		X		
Bizarre behaviour (e.g., inappropriate giggling/laughter, talking to self, fixated staring) Clinical notes/instructions: Clinician-rated, based on observed behaviour	67%	22%	11%	78%	Discarded		X		
Things moving in slow motion or sped up	56%	33%	11%	67%	Discarded		X		
Feeling separated from what is happening around you (e.g., as if you are in the movie or a play)	63%	25%	13%	75%	Discarded		X		
Feeling disconnected from your own body or looking at things from outside your body	67%	33%	0%	67%	Discarded		X		
Colours seemed to be diminished in intensity or much brighter than you would have expected	67%	11%	22%	89%	Discarded		X		
Seeing things as if you were in a tunnel, or looking through a wide-angle photographic lens	56%	44%	0%	56%	Discarded		X		
Things seem to be taking much longer or much less time than expected (e.g., as if time is passing slowly/standing still or there is a lifetime in a moment)	67%	11%	22%	89%	Things seem to be taking much longer or much less time than expected (e.g., as if time is passing slowly/standing still or there is a lifetime in a moment)	X			
Unprompted inconsolable crying	56%	22%	22%	78%	Discarded		X		
Unprompted hysterical laughter	44%	33%	22%	67%	Discarded		X		
Afraid without being able to say exactly why	78%	11%	11%	89%	Discarded		X		

	Keep	Discard	Modify	Keep + Modify	OUTCOME	Keep	Discard	Modify	Add
Feeling isolated from everything and everyone	67%	22%	11%	78%	Discarded		X		
Body feeling numb, dead or weird	33%	33%	33%	67%	Discarded		X		
Difficulty making even the smallest decision	44%	44%	11%	56%	Discarded		X		
Thoughts and/or actions being slowed down or sped up	78%	11%	11%	89%	Thoughts and/or actions feeling slowed down or sped up	X			
