# Supplementary material for: Development of the MDMA-Assisted Psychotherapy Side Effects Tool (M-SET): a Delphi study
Source: BMJ Open. 2026 May 11;16(5):e105630. doi: 10.1136/bmjopen-2025-105630 (PMC13182389; doi:10.1136/bmjopen-2025-105630)
Supplement: Supplementary file 3 [file bmjopen-2025-105630.reviewer_comments.pdf]

PEER REVIEW HISTORY

BMJ Open publishes all reviews undertaken for accepted manuscripts. Reviewers are asked to complete a checklist review form and are provided with free text boxes to elaborate on their assessment. These free text comments are reproduced below.

ARTICLE DETAILS

Title (Provisional)

Development of the MDMA-Assisted Psychotherapy Side Effects Tool (M-SET): A Delphi Study

Authors

Colcott, Julia; Guerin, Alexandre A; Carter, Olivia; Baggott, Matthew J; Bershad, Anya; Danforth, Alicia; de Wit, H; Feduccia, Allison A; Kirkpatrick, Matthew G; Liechti, Matthias E; Oehen, Peter; Schmid, Yasmin; Bedi, Gillinder

---

VERSION 1 - REVIEW

Reviewer 1

Name Eaton, Erica

Affiliation Center for Alcohol and Addiction Studies, Brown University, Providence, RI, USA

Date 10-Sep-2025

COI None

This manuscript addresses an important gap in psychedelic-assisted therapy research: the absence of a structured tool to systematically assess side effects in MDMA-assisted psychotherapy (MDMA-AP). The authors describe the development of the M-SET using a Delphi consensus process with international experts. The topic is timely and highly relevant, making a meaningful contribution to the literature as MDMA-AP approaches regulatory approval. The study is well-conceived and methodologically rigorous. However, there are some questions regarding the scope, panel representation, and practical feasibility.

Overall, this is a valuable and innovative contribution to the field. I offer a few suggestions and revisions below that I hope will help improve the clarity and impact of the paper:

Introduction

• The authors convincingly convey the need for a standardized side effect tool, given reliance on spontaneous reporting in current trials, a main point made by the US FDA when they voted to not approve MDMA-AT for PTSD last Summer. The work is timely as MDMA-AP enters clinical practice in Australia, Canada, and Switzerland.

• The introduction could more explicitly discuss limitations of existing tools (e.g., Systematic Assessment for Treatment Emergent Events, general psychiatric side effect scales) and how M-SET improves upon them.

Method

• The Delphi panel included 12 experts, but it is not clear how diverse the perspectives were (e.g., therapists delivering MDMA-AP, patient advocates, regulators). Including voices beyond researchers could strengthen ecological validity.

• Approximately 14% of items did not reach consensus. While the authors note this, the final decisions appear to rely on investigator judgment, which may introduce bias. More transparency on how these decisions were made would be helpful for replication studies and the reader.

Results

• A table summarizing domains covered by the final M-SET would aid readability.

Discussion

• At 165 items, with 49 during acute sessions, the burden on participants (who may be in highly vulnerable states) is considerable. Without piloting, it is uncertain whether the tool is feasible in real-world clinical practice. Consider adding a brief discussion of how digital platforms (e.g., REDCap, tablets, mobile apps) might help mitigate concerns about tool length and participant burden. In addition, the authors could note in next steps the importance of exploration of shortened versions for routine clinical use.

• The M-SET has not yet undergone psychometric testing (reliability, validity, sensitivity). The lack of validation should be emphasized more strongly to prevent premature adoption.

**Reviewer**	**2**
**Name**	**Lueken, Ulrike**
**Affiliation**	**Humboldt-Universitat zu Berlin, Department of Psychology**
**Date**	**30-Dec-2025**
**COI**	**None**

The authors introduce the development process and results for an instrument assessing side effects of MDMA-assisted psychotherapy (M-SET). The development was guided by a Delphi process including experts in the field. Item development was further informed by the results of a systematic review and meta-analysis as well as the use of broad and widely accepted instruments in the field. The result is a comprehensive set of scales incorporating both mental and physical (side-) effects at different time-points which makes it very useful for different (research as well as clinical application) purposes.

This manuscript is very timely in light of the dynamic developments in the field of MDMA-AP. It will help to systematically collect unbiased evidence on potential side-effects and their frequency both in research and clinical settings. As such, it is a necessary step in safeguarding the clinical use of MDMA for both scientists, clinicians and patients.

While I do not have any major concerns regarding the development process or manuscript content, as a naïve reader some open questions came up regarding the format of the scales. I hope that my suggestions may improve the comprehensibility and hence applicability and interpretation of the scales.

Unfortunately, the two figures were not readable in the pdf format that was provided for review. I tried to get the necessary information from the text, but one may consider scaling down the figures for further review.

One minor comment also refers to the wording of “psychiatric disorders/symptoms” etc. Maybe the authors might consider using “mental disorders” as – to my understanding – the term “psychiatric” may rather refer to characteristics of psychiatrists…

Recommendations on the scale format:

M-SET-Screening:

- Relevant history and comorbidity: while for psychotic disorders it is asked both for active or past symptoms, it remains unclear whether this should also be applied to the subsequent conditions. Maybe add whether current or lifetime presence should be rated?

- Pregnancy: maybe consider whether (mode of) contraception should be assessed?

- History of MDMA use: “How did you take it (…) and in which form (…)?” maybe use check box format here also?

- History of MDMA use: I was wondering if patients’ answers may be biased if they think this could negatively affect study inclusion. Maybe add one sentence on the rationale (safety, confidentiality of information) in the instructions to increase the validity of reporting?

M-SET-Baseline:

- The instructions for the patient self-report are such that they should evaluate the occurrence of symptoms over the past week. In addition, they should state of symptoms occurred “never”. I tried to respond on my own experience and found it had to answer the questions in case that symptoms occurred, but not within the last week. Maybe consider add an option stating “yes, but not within the last week”?

M-Set Acute Treatment – Pre-Treatment:

- Drug Effects Questionnaire: as far as I understood the assessment points of the scale this has to be answered before the drug is actually given. Does it make sense to ask these questions if no drug is given (“Do you feel a drug effect right now?”). As a patient I would be slightly confused.

- Regarding the timing when the scale is actually applied: the MDMA dose timing is requested. However, maybe add instructions when the questionnaire is to be completed? To me it remained a bit unclear whether the scale is given after the effects have actually subsided. It could improve the validity of the results if this is actually specified ("please fill out the scale after the acute effects have subsided"). I do not think that the scale is meant to be completed under the acute influence of the drug, right?

M-Set Acute Treatment – Pre-Supp Dose (180 minutes):

- Again, it remained unclear to me when exactly this questionnaire should be applied. This may be due to my limited knowledge about the dose escalation regimens in MDMA-PA, but clarification could be helpful in improving the validity of responses.

- Drug Effect Questionnaire: again, when will this be filled out? May be important to interpret the results (acute, subacute).

M-Set – Follow-up:

- Maybe consider adding information on the time interval since dosing?

- I also felt that in order to avoid confusion about follow-up assessments maybe use the term "Post" here and add the possibility to use the scale for additional follow-ups (e.g., weeks or months after the dosing session)?

- Symptoms: "Never" -> since the dosing? Or add additional category "not since dosing session"?

VERSION 1 - AUTHOR RESPONSE

Reviewer: 1

This manuscript addresses an important gap in psychedelic-assisted therapy research: the absence of a structured tool to systematically assess side effects in MDMA-assisted psychotherapy (MDMA-AP). The authors describe the development of the M-SET using a Delphi consensus process with international experts. The topic is timely and highly relevant, making a meaningful contribution to the literature as MDMA-AP approaches regulatory approval. The study is well-conceived and methodologically rigorous.

We thank the reviewer for their positive review.

However, there are some questions regarding the scope, panel representation, and practical feasibility.

Overall, this is a valuable and innovative contribution to the field. I offer a few suggestions and revisions below that I hope will help improve the clarity and impact of the paper:

Introduction

• The authors convincingly convey the need for a standardized side effect tool, given reliance on spontaneous reporting in current trials, a main point made by the US FDA when they voted to not approve MDMA-AT for PTSD last Summer. The work is timely as MDMA-AP enters clinical practice in Australia, Canada, and Switzerland.

We thank the reviewer for highlighting the importance and timeliness of the development of the tool.

• The introduction could more explicitly discuss limitations of existing tools (e.g., Systematic Assessment for Treatment Emergent Events, general psychiatric side effect scales) and how M-SET improves upon them.

We have expanded on the limitations of existing tools in the Introduction, **Page 6** of the revised manuscript:

"This is of concern in terms of accurate characterization of the risk-benefit profile of MDMA-AP, given that MDMA-AP may have a unique side effect profile relative to other treatments, including mystical experiences, possible increased attachment to therapists, and changes in personality (Colcott et al., 2024; Meikle et al., 2023; Wagner et al., 2017). While other scales such as the Systematic Assessment for Treatment Emergent Events or psychiatric side effect scales have the capacity to assess general side effects, a more specific tool is needed to robustly measure and characterise MDMA-AP side effects during and after the course of treatment."

Method

• The Delphi panel included 12 experts, but it is not clear how diverse the perspectives were (e.g., therapists delivering MDMA-AP, patient advocates, regulators). Including voices beyond researchers could strengthen ecological validity.

Panel members included researchers and clinicians with significant involvement in research investigating the therapeutic use in clinical populations or acute administration of MDMA in healthy volunteers. Notably, the panel had expertise in both administering MDMA in experimental studies and delivering MDMA-Assisted Psychotherapy. We agree with the reviewer that involving patient advocates and other community groups with diverse perspective could strengthened the validity of the tool. Accordingly, we have now included the following in the limitations and future directions on **Page 16** of the revised manuscript:

"While we conducted a robust Delphi process with an expert panel of researchers and clinicians with significant involvement in administration of MDMA to humans and delivery of MDMA-AP, future research should aim to refine the M-SET with consultation from patient advocates, regulators, and other relevant stakeholder groups."

• Approximately 14% of items did not reach consensus. While the authors note this, the final decisions appear to rely on investigator judgment, which may introduce bias. More transparency on how these decisions were made would be helpful for replication studies and the reader.

We have included more information on how items that did not meet criteria were assessed on **Page 10** of the revised manuscript:

"Items that did not reach consensus in Round 2 but reached an 80% threshold when keep and modify responses were combined, were reviewed on a case-by-case basis by the core research team (JC, AAG, and GB), who assessed whether to include or discard these items via consensus. To reduce bias, the final tool was approved and endorsed by all panellists prior to submission for publication."

Results

• A table summarizing domains covered by the final M-SET would aid readability.

We have now added a Table with domains covered by each item in the four modules of the M-SET (see below).

Module	Relevant medical history	Relevant psychiatric history	Physical examination (e.g., Weight; Height)	Clinical investigations (e.g., bloods, ECG)	Physical and physiological symptoms	Psychological symptoms	Vital signs	Acute drug effects
Screening Questionnaire	✓	✓	✓	✓	✓	✓	✓	
Baseline Questionnaire					✓	✓		
Acute Questionnaire					✓	✓	✓	✓
Follow-Up Questionnaire					✓	✓	✓	

Discussion

• At 165 items, with 49 during acute sessions, the burden on participants (who may be in highly vulnerable states) is considerable. Without piloting, it is uncertain whether the tool is feasible in real-world clinical practice. Consider adding a brief discussion of how digital platforms (e.g., REDCap, tablets, mobile apps) might help mitigate concerns about tool length and participant burden. In addition, the authors could note in next steps the importance of exploration of shortened versions for routine clinical use.

We acknowledge that the M-SET is a lengthy tool, however we aimed for the initial iteration of the tool to be comprehensive, to ensure that it would fully characterise the safety profile of MDMA-AP. We anticipate that future iterations will be more streamlined. We have been using the measure in one of our clinical trials, and the Acute Questionnaire takes on average between 5 to 20 minutes to administer, depending on the number of symptoms endorsed. Notably, not all symptoms are participant-rated, with nine in the acute section assessed based on clinician observation. The use of tablets to collect data in our study also reduces participant burden. Lastly, this tool can be adapted for clinical and research use based on the needs of the researcher or clinician using it.

We have now clarified these points on **Page 16** of the revised manuscript:

"The M-SET acute questionnaire assesses 49 different symptoms at multiple time points on the day of medication sessions. This has the potential to impose a burden on participants. However, this is somewhat mitigated by the fact that some items use clinician ratings and observation rather than participant self-report, and some of the self-reported sections can be completed using digital technologies (e.g., tablets or mobile phones) to reduce participant burden. In addition, researchers or clinicians can adapt the timing and specific content of the questionnaire to suit their particular contexts. To further reduce burden on participants and patients, it is important to explore the development of shortened versions of the M-SET for routine clinical use".

• The M-SET has not yet undergone psychometric testing (reliability, validity, sensitivity). The lack of validation should be emphasized more strongly to prevent premature adoption.

We agree with the reviewer and have already noted this in the Limitations and recommendations. In addition, we have added the following text on **Page 16** of the revised manuscript:

“The MSET has not yet undergone reliability, validity and sensitivity testing, and it is recommended that the M-SET be tested and validated in healthy and clinical populations and refined over time”.

We have also included this as one of the points in the Strengths and limitations of this study (after the Abstract).

Review: 2

The authors introduce the development process and results for an instrument assessing side effects of MDMA-assisted psychotherapy (M-SET). The development was guided by a Delphi process including experts in the field. Item development was further informed by the results of a systematic review and meta-analysis as well as the use of broad and widely accepted instruments in the field. The result is a comprehensive set of scales incorporating both mental and physical (side-) effects at different time-points which makes it very useful for different (research as well as clinical application) purposes.

This manuscript is very timely in light of the dynamic developments in the field of MDMA-AP. It will help to systematically collect unbiased evidence on potential side-effects and their frequency both in research and clinical settings. As such, it is a necessary step in safeguarding the clinical use of MDMA for both scientists, clinicians and patients.

We thank the reviewer for their thorough review and positive comments.

While I do not have any major concerns regarding the development process or manuscript content, as a naïve reader some open questions came up regarding the format of the scales. I hope that my suggestions may improve the comprehensibility and hence applicability and interpretation of the scales.

Unfortunately, the two figures were not readable in the pdf format that was provided for review. I tried to get the necessary information from the text, but one may consider scaling down the figures for further review.

Apologies for this issue - we have revised and re-submitted the Figures.

One minor comment also refers to the wording of “psychiatric disorders/symptoms” etc. Maybe the authors might consider using “mental disorders” as – to my understanding – the term “psychiatric” may rather refer to characteristics of psychiatrists…

We have changed "psychiatric disorders" to "mental disorders" throughout the revised manuscript.

Recommendations on the scale format:

We thank the reviewer for their recommendations. We cannot add or remove items from the M-SET because it has already gone through the Delphi process and been endorsed by the expert panel. However, we have clarified items/questions based on the reviewer's comments whenever possible.

All changes and clarifications to the M-SET are tracked in the **Appendix**.

M-SET-Screening:

- Relevant history and comorbidity: while for psychotic disorders it is asked both for active or past symptoms, it remains unclear whether this should also be applied to the subsequent conditions. Maybe add whether current or lifetime presence should be rated?

We have clarified "Current or lifetime" for Items 1-4, 6-10, and 12-13, and "Current" for Item 5

- Pregnancy: maybe consider whether (mode of) contraception should be assessed?

We have added a free-text field to specify mode of contraception (if any) to Item 15.

- History of MDMA use: "How did you take it (...) and in which form (...)?" maybe use check box format here also?

We thank the reviewer the suggestion. We added tickboxes for the "How did you take" and "In what form" questions.

- History of MDMA use: I was wondering if patients' answers may be biased if they think this could negatively affect study inclusion. Maybe add one sentence on the rationale (safety, confidentiality of information) in the instructions to increase the validity of reporting?

We have intentionally left the instructions blank for this module as what the screening clinician will say to prospective patients/participants will depend on the patient and the context in which the module is administrated. It is expected that clinician will cover confidentiality prior to enrolment.

M-SET-Baseline:

- The instructions for the patient self-report are such that they should evaluate the occurrence of symptoms over the past week. In addition, they should state of symptoms occurred "never". I tried to respond on my own experience and found it had to answer the questions in case that symptoms occurred, but not within the last week. Maybe consider add an option stating "yes, but not within the last week"?

We thank the review for their suggestion. We have reworded the "Never" option to "Not in the past week".

M-Set Acute Treatment – Pre-Treatment:

- Drug Effects Questionnaire: as far as I understood the assessment points of the scale this has to be answered before the drug is actually given. Does it make sense to ask these questions if no drug is given ("Do you feel a drug effect right now?"). As a patient I would be slightly confused.

The Drug Effects Questionnaire (DEQ) is administered before any drug is given to teach the participant how to use the scale before receiving MDMA. It is expected that the participant will answer "Not At All" to every question in the Pre-Treatment questionnaire. We have clarified in the Pre-Treatment DEQ:

"If you have not yet taken any medication, please respond NOT AT ALL to the questions below"

- Regarding the timing when the scale is actually applied: the MDMA dose timing is requested. However, maybe add instructions when the questionnaire is to be completed? To me it remained a bit unclear whether the scale is given after the effects have actually subsided. It could improve the validity of the results if this is actually specified ("please fill out the scale after the acute effects have subsided"). I do not think that the scale is meant to be completed under the acute influence of the drug, right?

The M-SET Acute modules should be administered at regular intervals throughout the acute medication session to assess and track the course of side effects after MDMA administration. For example, in one of our ongoing studies we administer the M-SET Acute at 60 min, 180 min, and 240 min post-MDMA administration. Frequency of administration will vary based on the clinical or research context.

We have updated the M-SET forms to include a "M-SET Acute Treatment - X minutes Post-Treatment" to improve clarity around the timing.

The “M-SET Acute Treatment – End of Treatment” should be used to assess whether symptoms and side effects have resolved before safely discharging participants.

M-Set Acute Treatment – Pre-Supp Dose (180 minutes):
- Again, it remained unclear to me when exactly this questionnaire should be applied. This may be due to my limited knowledge about the dose escalation regimens in MDMA-PA, but clarification could be helpful in improving the validity of responses.

This is an optional module which can be used to support decision making around administration of a supplemental dose of MDMA during an acute drug administration session. It should be completed before administrating the supplemental dose to ensure the participant can tolerate a second dose within the same session. In our ongoing study, this is done at the 180 min mark, but exact timing will vary based on the clinical/research protocol.

We have removed the “180 min” in the header of the form for clarity.

- Drug Effect Questionnaire: again, when will this be filled out? May be important to interpret the results (acute, subacute).

The DEQ is administered after MDMA administration, during the acute phase.

M-Set – Follow-up:
- Maybe consider adding information on the time interval since dosing?

- I also felt that in order to avoid confusion about follow-up assessments maybe use the term “Post” here and add the possibility to use the scale for additional follow-ups (e.g., weeks or months after the dosing session)?

The Follow-Up module is meant to be administered at follow-up timepoints (i.e., on days/weeks/months following medication sessions). As per the other modules, the number or frequency of follow-ups is at the discretion of the clinicians/researchers using the M-SET, and will vary based on protocols.

- Symptoms: “Never” -> since the dosing? Or add additional category “not since dosing session”?

We thank the reviewer for their suggestion. We have reworded the “Never” option to “Not Since Last Medication Session”

VERSION 2 - REVIEW

**Reviewer**	**1**

Name **Eaton, Erica**

Affiliation **Center for Alcohol and Addiction Studies, Brown University, Providence, RI, USA**

Date **18-Feb-2026**

COI

Thank you to the authors for the thoughtful and thorough revisions. The manuscript is meaningfully strengthened in response to the prior feedback. The rationale for developing an MDMA-assisted psychotherapy–specific safety instrument is now more clearly articulated, particularly regarding the limitations of general adverse event reporting tools in this context. The expanded description of panel composition, clearer explanation of how non-consensus items were handled, added discussion of feasibility considerations, and inclusion of a domain summary table all improve the manuscript's clarity and methodological transparency. The limitations section appropriately acknowledges the need for psychometric validation, feasibility testing, and broader stakeholder engagement in future iterations.

I would suggest one additional refinement. In the Clinical Implications section, the statement that "The M-SET is recommended for use in both research and clinical settings" reads somewhat stronger than warranted at this stage, given the absence of formal reliability and validity data, feasibility testing, and patient input. Softening the recommendation for clinical implementation until these data are available would better align with best practices in instrument development and with the limitations the authors thoughtfully outline.

Reviewer **2**

Name **Lueken, Ulrike**

Affiliation **Humboldt-Universitat zu Berlin, Department of Psychology**

Date **18-Feb-2026**

COI

The authors have adequately addressed my comments. I do noit have additional comments.
